# *Sinorhizobium meliloti* Sigma Factors RpoE1 and RpoE4 Are Activated in Stationary Phase in Response to Sulfite

**DOI:** 10.1371/journal.pone.0050768

**Published:** 2012-11-30

**Authors:** Bénédicte Bastiat, Laurent Sauviac, Carole Picheraux, Michel Rossignol, Claude Bruand

**Affiliations:** 1 INRA, Laboratoire des Interactions Plantes-Microorganismes, UMR441, Castanet-Tolosan, France; 2 Centre National de la Recherche Scientifique, Laboratoire des Interactions Plantes-Microorganismes, UMR2594, Castanet-Tolosan, France; 3 Fédération de Recherche 3450, Agrobiosciences, Interactions et Biodiversités, Plateforme Protéomique Génopole Toulouse Midi-Pyrénées, Institut de Pharmacologie et de Biologie Structurale, Centre National de la Recherche Scientifique, Toulouse, France; 4 Université Paul Sabatier, Université de Toulouse, Toulouse, France; Institute of Genetics and Molecular and Cellular Biology, France

## Abstract

Rhizobia are soil bacteria able to establish a nitrogen-fixing symbiosis with legume plants. Both in soil and *in planta*, rhizobia spend non-growing periods resembling the stationary phase of *in vitro*-cultured bacteria. The primary objective of this work was to better characterize gene regulation in this biologically relevant growth stage in *Sinorhizobium meliloti*. By a tap-tag/mass spectrometry approach, we identified five sigma factors co-purifying with the RNA polymerase in stationary phase: the general stress response regulator RpoE2, the heat shock sigma factor RpoH2, and three extra-cytoplasmic function sigma factors (RpoE1, RpoE3 and RpoE4) belonging to the poorly characterized ECF26 subgroup. We then showed that RpoE1 and RpoE4 i) are activated upon metabolism of sulfite-generating compounds (thiosulfate and taurine), ii) display overlapping regulatory activities, iii) govern a dedicated sulfite response by controlling expression of the sulfite dehydrogenase SorT, iv) are activated in stationary phase, likely as a result of endogenous sulfite generation during bacterial growth. We showed that SorT is required for optimal growth of *S. meliloti* in the presence of sulfite, suggesting that the response governed by RpoE1 and RpoE4 may be advantageous for bacteria in stationary phase either by providing a sulfite detoxification function or by contributing to energy production through sulfite respiration. This paper therefore reports the first characterization of ECF26 sigma factors, the first description of sigma factors involved in control of sulphur metabolism, and the first indication that endogenous sulfite may act as a signal for regulation of gene expression upon entry of bacteria in stationary phase.

## Introduction

Bacteria are exposed to many stressful conditions in nature, including nutrient starvation, which can limit their growth for long time periods. In laboratory conditions, starvation is mimicked by exhaustion of a growth-limiting element from the culture medium, most often the carbon source, and leads bacterial cells to transit from exponential growth to the so-called stationary phase, characterized by the absence of visible growth [1], [2]. Bacteria have evolved a number of different strategies that make them able to survive these famine periods. Those strategies mainly rely on global reorganization of gene expression, resulting in a number of morphological, physiological and metabolic changes, some of which confer multiple stress resistance to the cells and thus improve their ability to survive on the long term [2]. Among the regulators involved in this reprogramming are alternative sigma factors, which by associating with RNA polymerase (RNAP) change its specificity of promoter recognition. In exponential phase, the vegetative sigma factor is responsible for the transcription of housekeeping genes. At the onset of stationary phase, various signals and mechanisms make alternative sigma factors available for interacting with the core RNAP, thus allowing the holoenzyme to recognize new promoters and express new sets of genes. In *Escherichia coli*, the master general stress response regulator RpoS is the main sigma factor in charge of gene expression in stationary phase, as it controls the transcription of several hundreds of genes involved in functions as diverse as acquisition of multiple stress resistance, redirection of metabolism or structuration of the cell envelope [3], [4]. Other alternative sigma factors, such as the heat shock sigma factor RpoH or the extracytoplasmic function (ECF) sigma factor RpoE are also active at the end of exponential phase and upon entry in stationary phase, respectively, and mainly control the expression of chaperones and proteases involved in folding and degradation of cytoplasmic and secreted proteins, respectively [5], [6], [7].

*Sinorhizobium meliloti* is a Gram-negative bacterium belonging to the α-subclass of Proteobacteria. This soil bacterium can establish a symbiotic association with legume plants of the *Medicago* genera, including alfalfa (*M. sativa*) and the model legume *M. truncatula* (for a review see [8]). Soil is an oligotrophic environment where bacteria suffer nutrient starvation and thus spend most of their time in a stationary phase-like state [9], [10]. In symbiotic root organs (nodules) of legumes, most infecting bacteria as well as fully differentiated nitrogen-fixing bacteroids are non-growing bacterial forms [11], [12] whose transcriptional activity, as judged from whole genome analysis of gene expression, suggests that they are in a state resembling the stationary phase of free-living bacteria [13]. Transcriptomic analysis of global changes of gene expression in bacteria upon entry in stationary phase in laboratory conditions showed deep modifications, including up-regulation of hundreds of genes in comparison with exponentially growing bacteria, suggesting the involvement of several transcriptional activators [13], [14], [15]. Nevertheless, little is known about the nature of these regulators, the signals leading to their activation or the genes they control.

*S. meliloti* does not encode any RpoS homologue. In contrast, 14 alternative sigma factors, including RpoN, two heat shock sigma factors (RpoH1 and RpoH2) and eleven ECF sigma factors (RpoE1-11) are present in *S. meliloti* [16]. The role of RpoN in the transcriptional control of genes involved in nitrogen fixation, transport of dicarboxylic acids and nitrogen assimilation is well established in rhizobia [17], [18], [19]. *rpoH1* and *rpoH2* are transcriptionally up-regulated after a heat shock as well as in stationary phase [20]. RpoH1 is needed for growth at high temperature or at low pH, as well as for efficient symbiotic nitrogen fixation [20], [21], [22], [23]. Genes controlled by RpoH1 under acidic or heat shock conditions were determined and include chaperone- and protease-encoding genes [23], [24]. The role of RpoH2 is less clear as single mutants do not display any phenotype, and RpoH2 does not participate significantly in the transcriptional response to heat shock [24]. Nevertheless, double *rpoH1 rpoH2* mutants have a strong symbiotic phenotype (absence of root nodules) which suggests that both sigma factors may have partly redundant functions [22], [25]. Accordingly, both RpoH1 and RpoH2 were shown to contribute to gene expression in stationary phase, and to share some target genes [24]. Among the ECF sigma factors, only RpoE2 has been characterized in detail, and is considered as a functional equivalent of *E. coli* RpoS for the regulation of the general stress response [14], [26]. Indeed, RpoE2 is activated in a number of stress conditions including entry in stationary phase, and controls the transcription of at least 45 genes, including other transcriptional regulators, such as *rpoH2*, as well as genes involved in stress resistance [14], [27], [28]. Accordingly, *rpoE2* mutants were found to be more sensitive than the wild type strain to multiple stresses [27], [28], [29], [30], although no symbiotic deficiency could be detected [14], [29].

Although RpoE2 is an important regulator of gene expression during the log to stationary phase transition, a number of genes up-regulated in stationary phase are not under RpoE2 control, which suggests that other regulators remain to be found [14]. The objective of this work was to explore the possibility that other sigma factors, in addition to RpoE2, are among these regulators. Using a combined tandem affinity purification/mass spectrometry approach, we identified RpoH2, RpoE1, RpoE2, RpoE3 and RpoE4 as possible interactors of the RNAP in stationary phase, which suggests that these sigma factors may be active under this condition. According to a recent classification RpoE1, RpoE3 and RpoE4 belong to the same subgroup (known as ECF26) of ECF sigma factors, which are poorly characterized [31]. RpoE1 and RpoE4 were further studied here and suggested to be directly or indirectly activated by endogenous generation of sulfite, either naturally upon entry in stationary phase, or upon metabolism of exogenously added sulfonated compounds. Interestingly, RpoE4 controls a response required for efficient growth in the presence of sulfite, suggesting that it may be advantageous for bacteria in stationary phase by providing either a sulfite detoxification function or an energy input through sulfite respiration.

## Materials and Methods

### Bacterial Strains and Growth Conditions

The strains used in this study are listed in Table 1. *Escherichia coli* strains were grown in Luria-Bertani (LB) medium at 37°C. *S. meliloti* strains were grown at 28°C, either in LB medium supplemented with 2.5 mM CaCl_2_ and 2.5 mM MgCl_2_ (LBMC; strain constructions and precultures), or in Vincent minimal medium (VMM; 7.35 mM KH_2_PO_4_, 5.74 mM K_2_HPO_4_, 1 mM MgSO_4_, 18.7 mM NH_4_Cl, 456 µM CaCl_2_, 35 µM FeCl_3_, 4 µM biotine, 48.5 µM H_3_BO_3_, 10 µM MnSO_4_, 1 µM ZnSO_4_, 0.5 µM CuSO_4_, 0.27 µM CoCl_2_, 0.5 µM NaMoO_4_; pH = 7) containing either 10 mM sodium succinate (VMMS), or 100 mM taurine (liquid VMMT) or 20 mM taurine (solid VMMT) as carbon sources. When required, antibiotics were added to these final concentrations: 100 to 300 µg ml^−1^ of streptomycin (Sm), 5 to 10 µg ml^−1^ tetracycline (Tc), 40 µg ml^−1^ gentamycin (Gm), 150 to 600 µg ml^−1^ trimethoprim (Tmp), 40 µg ml^−1^ hygromycin (Hyg), 50 µg ml^−1^ kanamycin (Kan), 50 µg ml^−1^ carbenicillin (Cb).

**Table 1:** Strains and plasmids used in this study.

Strain or plasmid	Description	Reference
***Sinorhizobium meliloti***		
GMI11495	Wt (2011 Sm^R^)	[66]
CBT997	Δ*rpoE4*	this study
CBT1022	Δ*rpoE1*	this study
CBT1064	Δ*rpoE1* Δ*rpoE4*	this study
CBT1267	Δ*sorT*	this study
CBT1333	Δ*rpoE4* Δ*sorT*	this study
CBT1183	P*rpoE1*:: pTH1703-P*rpoE1-lacZ*	this study
CBT1185	P*rpoE1*:: pTH1703-P*rpoE1-lacZ* Δ*rpoE1*	this study
CBT1191	P*rpoE1*:: pTH1703-P*rpoE1-lacZ* Δ*rpoE4*	this study
CBT1247	P*rpoE1*:: pTH1703-P*rpoE1-lacZ* Δ*rpoE1* Δ*rpoE4*	this study
CBT1315	P*rpoE1*:: pTH1703-P*rpoE1-lacZ* Δ*sorT*	this study
CBT1350	P*rpoE1*:: pTH1703-P*rpoE1-lacZ* Δ*sorT* Δ*rpoE1*	this study
CBT1354	P*rpoE1*:: pTH1703-P*rpoE1-lacZ* Δ*sorT* Δ*rpoE4*	this study
CBT1358	P*rpoE1*:: pTH1703-P*rpoE1-lacZ* Δ*sorT* Δ*rpoE1* Δ*rpoE4*	this study
CBT1218	P*rpoE4*:: pTH1703-P*rpoE4-lacZ*	this study
CBT1220	P*rpoE4*:: pTH1703-P*rpoE4-lacZ* Δ*rpoE1*	this study
CBT1224	P*rpoE4*:: pTH1703-P*rpoE4-lacZ* Δ*rpoE4*	this study
CBT1251	P*rpoE4*:: pTH1703-P*rpoE4-lacZ* Δ*rpoE1* Δ*rpoE4*	this study
CBT1317	P*rpoE4*:: pTH1703-P*rpoE4-lacZ* Δ*sorT*	this study
CBT1352	P*rpoE4*:: pTH1703-P*rpoE4-lacZ* Δ*sorT* Δ*rpoE1*	this study
CBT1356	P*rpoE4*:: pTH1703-P*rpoE4-lacZ* Δ*sorT* Δ*rpoE4*	this study
CBT1360	P*rpoE4*:: pTH1703-P*rpoE4-lacZ* Δ*sorT* Δ*rpoE1* Δ*rpoE4*	this study
***Escherichia coli***		
DH5α	*supE44* Δ*lacU169* (Φ80*dlacZ* ΔM15) *hsdR17 recA1 endA1 gyrA96 thi-1 relA1*	Invitrogen
**Plasmids**		
pMLBAD	Expression vector, inducible by arabinose (Tmp^R^)	[67]
pGEM-T	Cloning vector (Amp^R^)	Promega
pTH1703	Gene inactivation vector, *rfp-gus*, *gfp-lacZ* (Gm^R^)	[68]
pJQ200KS	Gene replacement vector (Gm^R^)	[69]
pJQ200mp19	Gene replacement vector (Gm^R^)	[69]
pRK2013	Helper plasmid for triparental matings (Kan^R^)	[70]
pBB56.1	pMLBAD-*rpoE1*	this study
pBB60.1	pMLBAD-*rpoE4*	this study
pBB79.1	pMLBAD-*sorT*	this study
pBB72.1	pTH1703-P*rpoE1-lacZ*	this study
pBB74.4	pTH1703-P*rpoE4-lacZ*	this study
pBB67.2	pJQ200mp19-Δ*rpoE1*	this study
pBB61.4	pJQ200mp19-Δ*rpoE4*	this study
pBB77.7	pJQ200mp19-Δ*sorT*	this study

To perform growth curves, overnight precultures grown in 5 ml LBMC medium supplemented with 100 µg ml^−1^ Sm were diluted to OD_600_ = 0.05−0.1 in 10–20 ml VMMS supplemented with 100 µg ml^−1 ^Sm, and grown for 6–8 h. Cells were again diluted in fresh VMMS (OD_600_ = 0.002), or VMMT (OD_600_ = 0.1) supplemented with Sm, and growth was measured by monitoring OD_600_ over several days. To test the ability of wt and mutant strains carrying pMLBAD derivatives to grow in the presence of taurine as sole carbon source, overnight precultures grown in 7 ml LBMC supplemented with Sm and 600 µg ml^−1^ Tmp were harvested by centrifugation and resuspended as above in either VMMS or VMMT supplemented with 100 µg ml^−1 ^Sm and 150 µg ml^−1^ Tmp.

### Strain and Plasmid Constructions

All plasmid constructions were performed in *E. coli* DH5α. Oligonucleotides used for PCR amplifications are listed in Table S1. Absence of mutations in all constructs was checked by DNA sequencing. ORFs and promoter fragments were amplified by PCR using *S. meliloti* genomic DNA as template and oligonucleotides listed in Table S1 as primers, and cloned into pGEM-T.

pMLBAD-*rpoE1* and pMLBAD-*rpoE4* plasmids were constructed by subcloning in pMLBAD an *Eco*RI/*Hin*dIII fragment from pGEMT-*rpoE1* or pGEMT-*rpoE4*, containing the *rpoE1* or *rpoE4* ORFs, respectively.

pTH1703-P*rpoE1-lacZ* and pTH1703-P*rpoE4-lacZ* derivatives were obtained by subcloning in pTH1703 a 380 bp *Sph*I/*Nsi*I or 480 bp *Xho*I/*Nsi*I fragment from pGEMT-P*rpoE1* or pGEMT-P*rpoE4*, containing the P*rpoE1* and P*rpoE4* promoters, respectively.

Chromosomal genes were deleted using pJQ200mp19 derivatives containing ∼400 bp regions flanking the gene to be deleted (*rpoE1*, *rpoE4* or *sorT*). These flanking regions were first produced by PCR using *S. meliloti* genomic DNA as template and oligonucleotides listed in Table S1 as primers, and were individually cloned into pGEM-T. They were subsequently juxtaposed as *Sal*I-*Nsi*I and *Nsi*I-*Xma*I fragments into *Sal*I/*Xma*I-cut pJQ200mp19.

Plasmids, either integrative or replicative, were introduced in the *S. meliloti* strain GMI11495 by triparental mating [32] using pRK2013 as a helper, with subsequent selection for antibiotic resistance. For the construction of deletion mutants, single-crossover genomic integration of the corresponding pJQ200 derivatives was selected for Gm resistance. Resulting strains were then propagated in absence of antibiotic, and cells having lost the plasmid by a second recombination event were selected by plating on LBMC supplemented with 5% sucrose (Suc). Suc^R^ Gm^S^ colonies were then screened by PCR analysis using as primers OCB935-OCB939, OCB942-OCB943 and OCB995-OCB1004 for deletion of *rpoE1*, *rpoE4* and *sorT*, respectively. For the construction of P*rpoE1*-*lacZ* and P*rpoE4-lacZ* reporter strains, single-crossover genomic integration (at the allelic position) of the corresponding pTH1703 derivatives was selected for Gm resistance, and correct location of the plasmids was checked by PCR using as primers OCB1042-lacPCR and/or GUS1-OCB991 (for *rpoE1-lacZ*) and OCB942-lacPCR and/or GUS1-OCB631 (for *rpoE4-lacZ*).

### DNA Sequencing of the SMc01420-1421 Region

The DNA sequence of the SMc01420-1421 region was determined as follows. A ∼670 pb fragment encompassing the SMc01420-SMc01421 junction was PCR-amplified using oligonucleotides OCB911 and OCB935 as primers, and genomic DNA of strains GMI11495 or 1021 [33] as template. The generated fragments were cloned into pGEM-T, and the DNA sequence of the inserts was determined using universal primers.

### Sulfite Assay

Sulfite was assayed using a fuchsin-based method [34]. Briefly, cultures were centrifuged in microtubes and 2 ml of culture supernatant, or a dilution of it, were mixed with 400 µl of freshly prepared fuchsin reagent (0.56 M H_2_SO_4_, 0.016% basic fuchsin, 0.16% paraformaldehyde) and incubated for 15 min at room temperature before OD_580_ measurement. Sulfite concentration was deduced from comparison with a range of standards (1-24 µM sulfite) prepared from a fresh 600 mg l^−1^ solution of sodium sulfite and tested in parallel. In this range, the sulfite determination was linear, with a lower detection limit ∼1–1.5 µM.

### Preparation of Samples for Microarrays, qRT-PCR, and β-galactosidase Assays

For microarray or quantitative reverse transcription-PCR (qRT-PCR) studies of *rpoE1* or *rpoE4* overexpression, overnight precultures (5 to 10 ml) in LBMC (supplemented with Sm and Tmp) of *S. meliloti* strains carrying pMLBAD derivatives were diluted to OD_600_ = 0.1 in 10 ml of VMMS and grown for 6 to 8 h. Cultures were then diluted once again in VMMS (130–150 ml) in order to reach OD_600_∼0.3–0.4 the day after. Arabinose was added to a final concentration of 0.2% to induce *rpoE1* or *rpoE4* expression and cultures were incubated a further 1h30. Several 20 ml aliquots of the cultures were then harvested by filtration, frozen in liquid nitrogen and stored at −80°C until use.

For other microarray or qRT-PCR or β-galactosidase analyses, overnight precultures (5 to 10 ml) in LBMC of *S. meliloti* strains were diluted to OD_600_ = 0.1 in 20 ml of VMMS and grown for 6 to 8 h. Cultures were then diluted once again in either VMMS (250 ml for RNA preparations or 20–30 ml for β-galactosidase assays), or centrifuged and resuspended in VMMT (150 ml for RNA preparations or 10–15 ml for β-galactosidase assays). When cultures in VMMS reached OD_600_∼0.1–0.2 the day after, they were divided in two flasks: one was kept without treatment, whereas the other was supplemented with 20 mM thiosulfate. After 2 h (for RNA preparations) or 4 to 24–30 h (for β-galactosidase assays), 20 ml of culture (for RNA preparations) were harvested by filtration, immediately frozen in liquid nitrogen and stored at −80°C, or 100 µl were collected for β-galactosidase assays. Cultures in VMMT were grown until OD_600_∼0.2 the day after and 25 ml were collected and treated as above for RNA preparation, or 100 µl were collected for β-galactosidase assays.

RNA was prepared from the collected samples as previously described [14], followed by DNase I treatment (QIAGEN clean-up procedure). β-galactosidase activity was assayed in the collected samples as described [35].

### Quantitative RT-PCR Analyses

Reverse transcription was performed using Superscript II reverse transcriptase (Invitrogen) with random hexamers as primers. RNA samples isolated from at least three independent experiments were tested for each condition. Real-time PCRs were run on a LightCycler system (Roche) using the FastStart DNA MasterPLUS SYBRGreen I kit (Roche) according to the manufacturer’s instructions. 16S rRNA was used as a reference for normalization using oligonucleotides OCB794–OCB796.

### Labeling of Hybridization Probes, Microarray Hybridizations, and Analyses

Cy3- and Cy5-labeled cDNAs were prepared according to the method of DeRisi and associates [36] from 15µg of RNA isolated from at least three independent experiments for each condition. For each of these three experiments, either one (Table 2, iii) or two (Table 2, i and ii) hybridizations were performed. Hybridizations were carried out as described previously [13], using Sm14koligo microarrays purchased from A. Becker (University of Bielefeld, Bielefeld, Germany). Data were acquired on GenePix 4000 (Axon Instruments) or Innoscan 900 (Innopsys) scanners, and quantifications of mean signal intensities for each spot were performed using GenePix Pro 3.0 (Axon Instruments). Data analyses were carried out using Genesight 3.5 (Biodiscovery). Data were normalized using the mean of the signals. Complete datasets are shown in Table S2, and have been submitted to the ArrayExpress database under the accession numbers E-MEXP-3471, E-MEXP-3472 and E-MEXP-3475.

**Table 2:** Microarray identification and qRT-PCR validation of *S. meliloti* genes controlled by RpoE1 and/or RpoE4.

		Fold-induction	
Gene^a^	Description	Microarrays^b^	qRT-PCR^c^
***i)*** ** rpoE1 ** ***over-expression (pMLBAD-*** **rpoE1 vs ** ***pMLBAD)***			
SMc01418	Hypothetical signal peptide protein	12.5	78.4
SMc01419 (*rpoE1*)	Putative ECF sigma factor	11.1	195.9
SMc01420	Putative anti-sigma factor	3.2	30.7
SMc01421	Hypothetical protein	2.8	ND
SMc02156	Conserved hypothetical protein	5.1	46.0
SMc04051 (*rpoE4*)	Putative ECF sigma factor	1.96	6.0
***ii)*** ** rpoE4 ** ***over-expression (pMLBAD-*** **rpoE4 vs ** ***pMLBAD)***			
**SMc04051 (** ***rpoE4*** **)**	Putative ECF sigma factor	10.9	98.8
**SMc04050**	Putative anti-sigma factor	-	24.7
**SMc04049 (** ***sorT*** **)**	Sulfite oxidase	3.7	43.9
**SMc04048**	Putative cytochrome *c*	3.5	ND
**SMc04047 (** ***azu2*** **)**	Probable pseudoazurin (blue copper protein)	2.2	ND
SMc04164	Conserved hypothetical protein	5.9	9.8
SMc00821	Conserved hypothetical protein	2.2	18.0
SMc00108	Putative acetyltransferase	3.9	18.9
***iii)*** ** rpoE4 ** ***deletion*** ** (** ***wt*** ** vs ΔrpoE4) ** ***in the presence of thiosulfate***			
**SMc04051 (** ***rpoE4*** **)**	Putative ECF sigma factor	10.8	NA
**SMc04050**	Putative anti-sigma factor	3.9	32.6
**SMc04049 (** ***sorT*** **)**	Sulfite oxidase	19.2	266.1
**SMc04048**	Putative cytochrome *c*	15.3	ND
**SMc04047 (** ***azu2*** **)**	Probable pseudoazurin (blue copper protein)	4.4	ND
SMb21671	Hypothetical protein	2.5	2.8

a Genes found to be regulated by RpoE4 in both experiments ii) and iii) are indicated in bold.

b All genes with ratio >2 and P value (t test) <0.05 in microarrays are shown, except SMc04051 which is included in i) because it is of interest for the study, and SMc04050 which did not show up in ii).

c All genes tested were significantly up-regulated (>2-fold, P<0.05), except SMb21671 in (iii) (P = 0.36).

ND, not determined. NA, not applicable.

### 5′ RACE Mapping of Transcription Start Sites

To map transcription start sites**,** we performed rapid amplification of cDNA 5′ ends (5′-RACE) as previously described [26]. Total RNA was prepared from GMI11495 cells grown with either succinate or taurine as carbon source, and 2 µg of RNA was used for reverse transcription for 1 hr at 42°C in the presence of Superscript II reverse transcriptase (Invitrogen) and using random hexamers as primers. As control, the same reaction was performed without addition of enzyme. Then, RNA templates were degraded with RNaseH, and cDNAs were purified on MicroSpin S-400 HR columns (GE Healthcare). 3′ ends of cDNAs were ligated with the anchor oligonucleotide DT88 by overnight incubation at 18°C in the presence of T4 RNA ligase (Promega). PCR were performed on aliquots of the ligation mixtures using DT89 and primers OCB951, OCB967, OCB983 and OCB971 specific for SMc01418, SMc02156, *rpoE4* and *sorT*, respectively. Amplification products were analysed by agarose gel electrophoresis. A DNA fragment larger than the distance from the primer to the translation start was obtained in each case, specific of the samples derived from the strain cultivated in the presence of taurine and treated with reverse transcriptase. This PCR product was gel-purified, cloned into pGEM-T and its sequence determined using universal primers.

### Plant Assays

Plant assays of symbiotic phenotypes were performed as previously described [37]. Briefly, seeds of *Medicago sativa* cv. Europe or *Medicago truncatula* Gaertn. cv. Jemalong A17 were surface sterilized, germinated on agar plates and allowed to grow on nitrogen-free Fahraeus medium in test tubes during three days. 11 to 27 plants were inoculated with ∼5.10^4^ bacteria/plant of each strain to be tested, in two independent experiments. The nodulation kinetics and aspect of the plants were followed for 40 days. The whole test was performed twice independently on *M. sativa* (35–38 plants) and once on *M. truncatula* (10–11 plants) with wt and Δ*rpoE1*, Δ*rpoE4* and Δ*rpoE1* Δ*rpoE4* mutant strains. The Δ*sorT* mutant was tested only once on *M. sativa* (26 plants).

## Results

### Determination of the RpoE1 and RpoE4 Regulons by Sigma Factor Overexpression

To identify *S. meliloti* sigma factors possibly activated in stationary phase, we pulled down the RNAP by a tap-tag approach, and identified co-purifying proteins by mass spectrometry (Information S1). Five alternative sigma factors were found among these proteins: the general stress response regulator RpoE2, the heat shock sigma factor RpoH2 and three extracytoplasmic function sigma factors (RpoE1, RpoE3 and RpoE4) belonging to the poorly characterized ECF26 subgroup. The following study focuses on RpoE1 and RpoE4.

**Figure 1:**
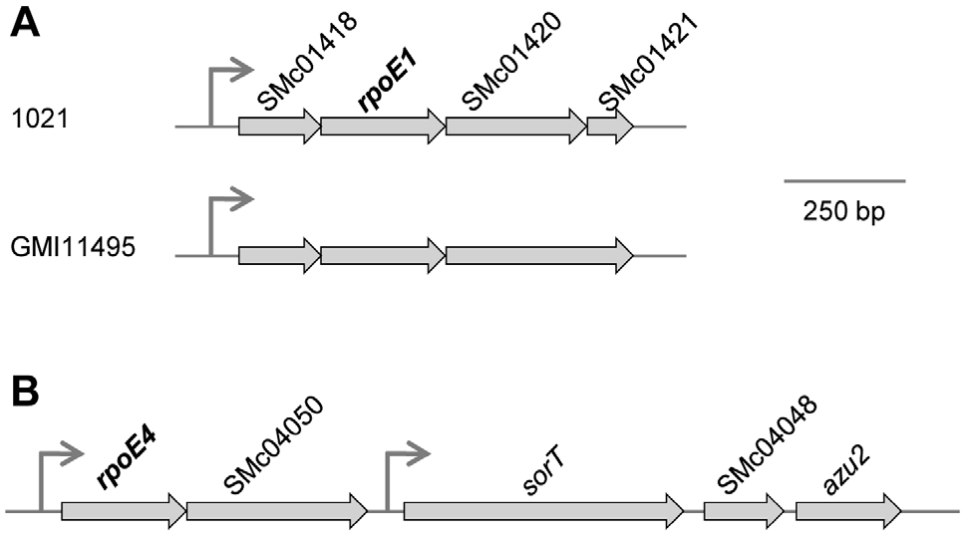
Schematic representation of the *rpoE1* (A) and *rpoE4* (B) chromosomal regions of *S. meliloti*. Grey-colored arrows represent open reading frames. Promoters mapped in the present study (Fig. 4) are indicated. In A is shown a comparison of the *rpoE1* regions in the reference strain 1021 and in strain GMI11495 used in this study (see text).

As a first step to understand the function of these sigma factors, we identified the genes directly or indirectly controlled by RpoE1, using microarrays. RpoE1 activation was mimicked in exponentially growing *S. meliloti* cells by over-expressing *rpoE1* (SMc01419) under control of the arabinose-dependent P_BAD_ promoter of plasmid pMLBAD-*rpoE1*. In the presence of arabinose, *S. meliloti* cells carrying this plasmid contained ∼200-fold as many *rpoE1* transcripts as control cells carrying the empty vector, as assessed by qRT-PCR (Table 2). Whole genome transcription profiles of these strains were compared using microarrays. In addition to *rpoE1*, 4 genes were up-regulated >2-fold (t test, P<0.05) upon *rpoE1* over-expression (Table 2), organised in two transcription units including the *rpoE1* operon (Fig. 1A). Induction of these genes was validated by qRT-PCR (Table 2). Interestingly, SMc04051 which encodes the putative ECF sigma factor RpoE4 was just below the threshold ratio used in our analysis (Table 2). qRT-PCR confirmed that *rpoE4* is up-regulated (6-fold) upon *rpoE1* over-expression (Table 2).

In the reference strain *S. meliloti* 1021, downstream of *rpoE1* are located two ORFs (SMc01420-01421) whose products display strong similarities with the N and C-terminal regions, respectively, of COG5662 domains, found in the majority of putative anti-sigma factors associated to ECF26 sigma factors [31] (Fig. 1A). Verification of the nucleotide sequence of this region in strains 1021 and GMI11495 used in the present study revealed a one-nucleotide difference (5 *vs* 6 G-stretch at position 607 of SMc01420 in 1021 *vs* GMI11495). Thus GMI11495 encodes a longer SMc01420 polypeptide with full-length similarity to anti-sigma factors (Fig. 1A), which is disrupted by a frame-shift in strain 1021. Strikingly, a previously published transcriptome comparison of strains 1021 and 2011 (the direct Sm^S^ ancestor of GMI11495) had revealed the six RpoE1 targets found here among the genes up-regulated in strain 1021 (see supplemental data in [38]). This could be explained if SMc01420-1421 encodes a partly inactivated RpoE1 anti-sigma factor in strain 1021, and further validates our determination of the RpoE1 regulon.

We also explored the RpoE4 regulon, using a similar approach based on *rpoE4* over-expression. In the presence of arabinose, the *S. meliloti* strain carrying pMLBAD-*rpoE4* grew more slowly than the control strain carrying the empty vector (doubling times ∼4 *vs* ∼3 hours, respectively), suggesting that *rpoE4* over-expression is, directly or indirectly, toxic to the cells. qRT-PCR analysis confirmed that *rpoE4* was over-expressed (∼100-fold) in this strain in comparison to the control strain carrying the empty vector (Table 2). Transcriptomic comparison of these strains using microarrays revealed 7 genes up-regulated upon *rpoE4* over-expression, in addition to *rpoE4* itself, and these data were validated by qRT-PCR (Table 2). The ORF SMc04050 located downstream of *rpoE4*, and supposed to form an operon with it (Fig. 1B) did not show up on microarrays, but was shown by qRT-PCR to be up-regulated upon *rpoE4* over-expression (Table 2). Moreover, RT-PCR analysis of the region using primers in both *rpoE4* and SMc04050 showed the existence of overlapping mRNA species, which confirms that they are indeed part of the same operon (not shown). *sorT* (SMc04049), SMc04048 and SMc04047, which are located just downstream of the *rpoE4-*SMc04050 operon (Fig. 1B) were previously shown to be transcribed as an operon [39]. Using RT-PCR, we were unable to detect any co-transcription of SMc04050 and *sorT* (data not shown), which suggests that these genes are part of two independent operons (Fig. 1B). Altogether, these data suggest that the sigma factors RpoE1 and RpoE4 control the expression of a few genes in addition to their own operons.

### RpoE4 and SorT are Required for Optimal *S. meliloti* Growth with Taurine as Sole Carbon Source

**Figure 2:**
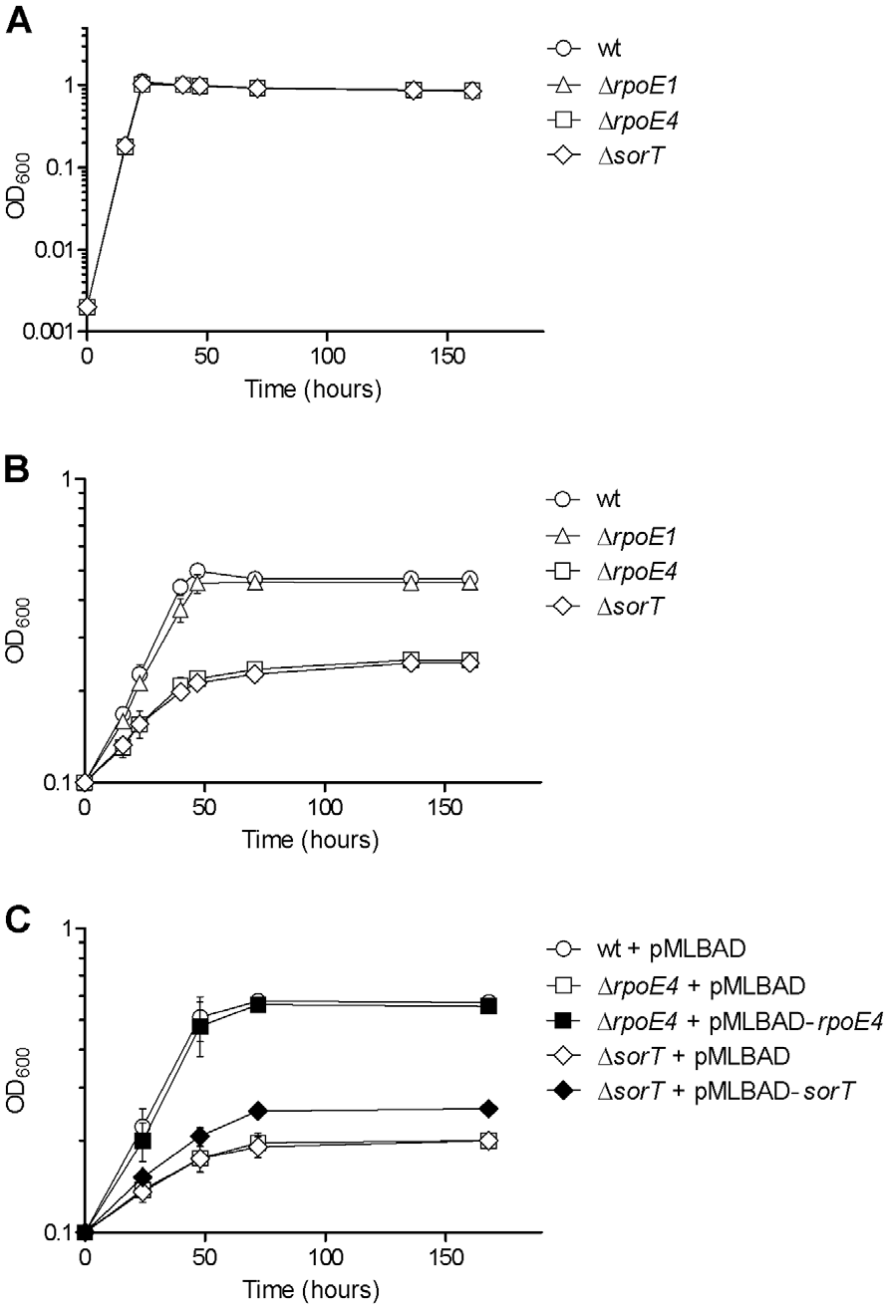
Growth curves of various *S. meliloti* strains, in the presence of succinate or taurine. Strains were cultured in Vincent minimal medium supplemented with either 10 mM sodium succinate (VMMS, A) or 100 mM taurine (VMMT, B and C) as sole carbone source. Strains GMI11495 (wt), CBT1022 (Δ*rpoE1*), CBT997 (Δ*rpoE4*), and CBT1267 (Δ*sorT*), carrying or not pMLBAD derivatives, as indicated, were pre-cultured to mid-log phase in VMMS, and were then either diluted in fresh VMMS to OD_600_ = 0.002 or centrifuged and resuspended in VMMT to OD_600_ = 0.1. Growth was monitored by measuring OD_600_ over several days. All media were supplemented with Sm, and with Tmp when strains contained pMLBAD derivatives (C). All strains carrying pMLBAD derivatives displayed similar growth curves in VMMS (not shown). The results shown are the means and standard errors of data from three independent experiments.

*sorT*, one of the RpoE4-regulated genes, was recently described as encoding a periplasmic sulfite dehydrogenase which catalyzes the two-electron oxidation of sulfite into sulfate (SO_3_^2−^+H_2_O → SO_4_^2−^ +2H^+^+2e^−^), although its physiological role in *S. meliloti* was not clearly established [39]. Interestingly, *sorT* expression was shown to be induced by taurine (2-aminoethane sulfonic acid) and thiosulfate, two sulfite-generating compounds. To determine whether RpoE4 could be involved in this regulation, we constructed a Δ*rpoE4* mutant. This mutant grew as efficiently as the wt strain in minimal medium with sodium succinate as a carbon source either in liquid cultures or on plates. In contrast, its growth was severely impeded (both growth rate and final density, or colony size) in comparison to that of the wt strain when taurine was used as a carbon source (Fig. 2A, B and data not shown). As control, a Δ*rpoE1* mutant grew as efficiently as the wt strain in the presence of taurine (Fig. 2B). A normal growth of the Δ*rpoE4* mutant could be restored by complementation with the plasmid pMLBAD-*rpoE4* (Fig. 2C). The growth defect of the Δ*rpoE4* mutant did not result from an increased lethality since mutant cells displayed a similar viability as wt cells, as assessed by measuring plating efficiency on LBMC (not shown).

To determine whether the phenotype of the Δ*rpoE4* mutant could result from the lack of *sorT* expression, we also constructed a Δ*sorT* mutant. This strain displayed the same growth defect as the Δ*rpoE4* mutant (Fig. 2B). This defect could be complemented, although partially, by the plasmid pMLBAD-*sorT*, showing that it resulted from the absence of SorT (Fig. 2C; partial complementation could result from insufficient *sorT* expression, as suggested further below by gene expression analyses, or from polar effects of the *sorT* mutation on expression of downstream genes, which encode putative electron acceptors of the SorT-catalysed oxidation reaction; [40]). In addition to this similarity of Δ*rpoE4* and Δ*sorT* strains, a double Δ*rpoE4* Δ*sorT* mutant displayed a growth phenotype indistinguishable from the single mutants (not shown) indicating that the two mutations do not have cumulative effects and thus likely affect the same pathway. Altogether, these data show that SorT is required for optimal growth of *S. meliloti* on taurine, and suggest that RpoE4 is activated in this condition and up-regulates the expression of *sorT*.

As SorT catalyses the oxidation of sulfite into sulfate, its absence should result in sulfite accumulation. Indeed, as determined using a fuchsin-based assay, 22-fold more sulfite was present in culture supernatants of Δ*sorT* mutant cells grown in the presence of taurine (245±39 µM) in comparison to wt cells (11±6 µM). A similar sulfite accumulation was observed in supernatants of Δ*rpoE4* mutant cells. These results therefore confirm that SorT is involved in sulfite degradation.

### RpoE4 and RpoE1 are Strongly Activated in Exponential Phase in the Presence of Taurine and Display Overlapping Regulatory Activities

To confirm that RpoE4 is activated in the presence of taurine, we measured the expression of RpoE4 target genes in wt and Δ*rpoE4* mutant strains. In the following experiments, gene expression was measured either using qRT-PCR on the genes found above to be regulated by RpoE4 (*sorT*, *rpoE4,* SMc04050) or RpoE1 (SMc02156, SMc01418, *rpoE1*), or using transcriptional *lacZ* fusions to the *rpoE4* or *rpoE1* promoter.

**Figure 3:**
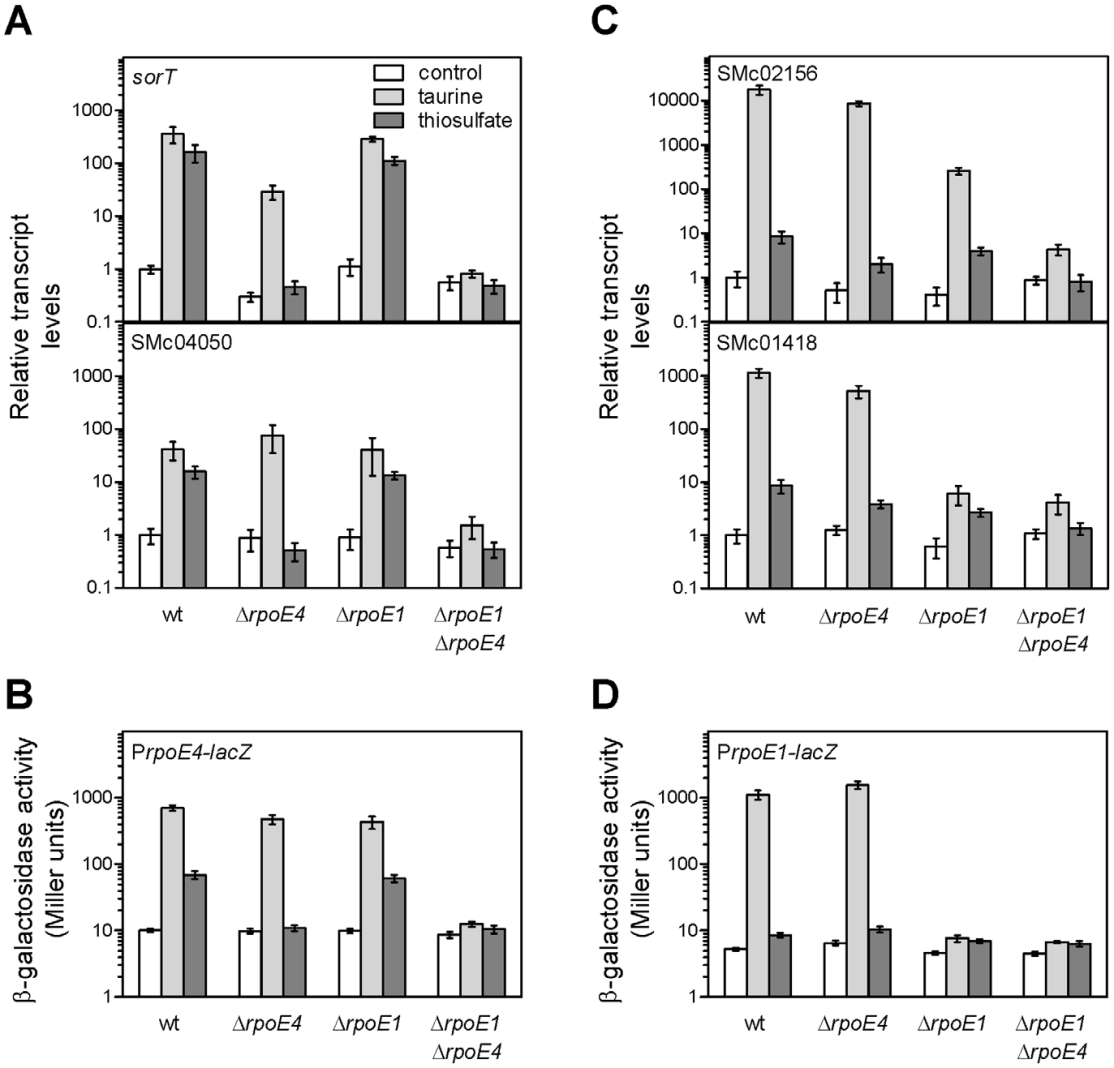
Expression levels of RpoE1 or RpoE4 target genes in the presence of sulfite-generating compounds, in various genetic backgrounds. A and C. Expression levels of *sorT*, SMc04050, SMc02156 and SMc01418 were measured by qRT-PCR from strains GMI11495 (wt), CBT997 (Δ*rpoE4*), CBT1022 (Δ*rpoE1*) and CBT1064 (Δ*rpoE1* Δ*rpoE4*), either grown with sodium succinate (white bars) or taurine (pale grey bars) as carbon source, or with succinate plus 20 mM thiosulfate (dark grey bars). Results are expressed as relative transcript levels, with wt control levels arbitrarily set to 1 for each gene, and are the means and standard errors of data from three to five independent experiments. B and D. Expression from the promoter of the *rpoE4* or *rpoE1* operon was estimated by measuring β-galactosidase activity driven from the chromosomal *PrpoE4-lacZ* fusion in strains CBT1218 (wt), CBT1224 (Δ*rpoE4*), CBT1220 (Δ*rpoE1*), and CBT1251 (Δ*rpoE1* Δ*rpoE4*), or from the chromosomal *PrpoE1-lacZ* fusion in strains CBT1183 (wt), CBT1191 (Δ*rpoE4*), CBT1185 (Δ*rpoE1*), and CBT1247 (Δ*rpoE1* Δ*rpoE4*), either grown with sodium succinate (white bars) or taurine (pale grey bars) as carbon source, or with succinate plus 20 mM thiosulfate (dark grey bars). The results shown are the means and standard errors of data from three to seven independent experiments.

The *sorT* and *rpoE4* operons were both up-regulated in the presence of taurine in the wt strain (Fig. 3A, B, Fig. S1). In the Δ*rpoE4* mutant, *sorT* expression was reduced (∼12-fold), but surprisingly remained significantly induced in comparison to the control (Fig. 3A). Similarly, expression of the *rpoE4* operon was still significantly activated by the presence of taurine in a Δ*rpoE4* mutant background (Fig. 3A, B). These data therefore suggest that RpoE4 is not the only sigma factor controlling expression of *sorT* and *rpoE4* operons. We therefore tested expression of the *rpoE4* and *sorT* operons in a Δ*rpoE1* mutant and in a double Δ*rpoE1* Δ*rpoE4* mutant. Induction of the *rpoE4* and *sorT* operons by taurine was not significantly affected in the single Δ*rpoE1* mutant, but was completely abolished in the double Δ*rpoE1* Δ*rpoE4* mutant (Fig. 3A, B). These results show that both RpoE4 and RpoE1 are activated by taurine in exponential phase, and contribute to *sorT* and *rpoE4* transcription. Nevertheless, the contribution of RpoE1 to *sorT* expression was apparently not enough to complement the growth defect of the *rpoE4* mutant on taurine (Fig. 2B).

That RpoE1 is activated in the presence of taurine was confirmed by showing that RpoE1 targets (SMc02156 and the *rpoE1* operon) are strongly up-regulated in cells grown on taurine (Fig. 3C, D, Fig. S1). Whereas this induction was unaffected in a Δ*rpoE4* mutant background, it was severely reduced (SMc02156) or almost abolished (*rpoE1* operon) in the Δ*rpoE1* mutant (Fig. 3C, D). This result therefore confirms that RpoE1 is activated in the presence of taurine. However, SMc02156 was still significantly induced in the Δ*rpoE1* mutant (Fig. 3C). This residual induction almost completely disappeared in the double Δ*rpoE1* Δ*rpoE4* mutant, indicating that RpoE4 also participates in the control of SMc02156 transcription. Note that SMc02156 was still weakly (∼5-fold) induced by taurine in the Δ*rpoE1* Δ*rpoE4* mutant, suggesting a possible control of these genes by other sigma factors.

**Figure 4:**
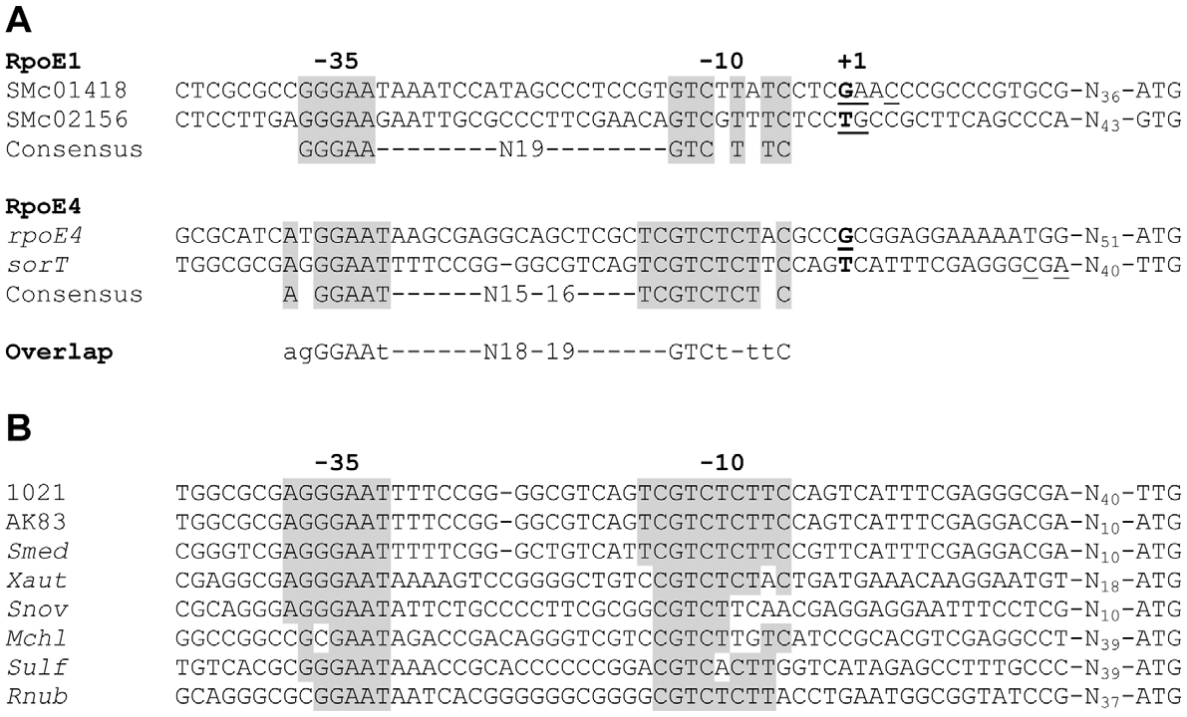
Promoter regions of various genes. A. Transcription start sites (+1) in promoter regions of *S. meliloti* genes controlled by RpoE1 and/or RpoE4, as determined from 5′RACE experiments in the present study (underlined) or deduced from Illumina- or 454-based RNAseq analyses (bold; [41]; Brice Roux, unpublished results). The deduced −10 and −35 sequences recognized by the sigma factors are highlighted in grey. Distance (in nucleotides) to the predicted translation start site of each ORF is indicated. B. The 5′ untranslated region of genes encoding known or putative sulfite-oxidizing enzymes from *S. meliloti* AK83, *S. medicae* WSM419 (*Smed*), *Xanthobacter autotrophicus* Py2 (*Xaut;* two identical sequences), *Starkeya novella* DSM506 (*Snov*), *Methylobacterium chloromethanicum* (*Mchl*), *Sulfitobacter* sp. EE-36 (*Sulf*; identical sequence in *Sulfitobacter* sp. NAS-14.1) and *Roseovarius nubinhibens* ISM (*Rnub*) are aligned with the *sorT* promoter region of *S. meliloti* 1021. Conserved putative −10 and −35 regions are highlighted in grey. See Fig. S4 for further details.

To understand the molecular bases of the overlapping regulatory activities of RpoE1 and RpoE4, the transcription start sites of SMc02156 and the *rpoE1*, *rpoE4* and *sorT* operons were determined by 5′RACE in wt cells grown on taurine as carbon source. Transcription start sites were also deduced from the position of transcript 5′ends determined in independent studies from Illumina- and 454-based RNA seq analyses of *S. meliloti* grown in various conditions ([41]; B. Roux, unpublished). Results are summarized in Fig. 4A. Although the low number of genes regulated by RpoE1 and RpoE4 made difficult to establish reliable consensus sequences, −35 and −10 regions recognized by RpoE1 or RpoE4 could be distinguished (Fig. 4A). They contain GAA and GT motifs often found in −35 and −10 regions of promoters controlled by ECF sigma factors [31]. Significant sequence similarities were found between the promoters recognized by RpoE1 and RpoE4 (Fig. 4A), which probably account for the cross-talks described above.

### RpoE4 and to a Lesser Extent RpoE1 are Activated in Exponential Phase in the Presence of Thiosulfate

As *sorT* was described as also up-regulated in the presence of thiosulfate [39], we measured the expression of RpoE4 target genes in wt and Δ*rpoE4* mutant strains under this condition. Bacteria grown to mid-log phase in minimal medium were exposed to 20 mM sodium thiosulfate. Thiosulfate addition led to a transient growth arrest of the cultures, but no difference was observed between wt and mutant cells which all recovered a normal growth rate after ∼2–3 hours. The expression level of the *sorT* and *rpoE4* operons was measured as described above, after two hours (qRT-PCR) or four hours (β-galactosidase) in either the presence or absence of thiosulfate (Fig. 3A, B, Fig. S1). The *sorT* and *rpoE4* operons were both up-regulated in the presence of thiosulfate in the wt strain, although at lower levels than in the presence of taurine. They were no longer induced by thiosulfate in the Δ*rpoE4* mutant (Fig. 3A, B), which shows that RpoE4 is activated in the presence of thiosulfate.

Surprisingly, RpoE1 was not able to complement the lack of RpoE4 in the presence of thiosulfate, whereas it was in the presence of taurine (see above). qRT-PCR on the RpoE1 targets *rpoE1*, SMc01418, and SMc02156 showed that they were weakly (∼4–9-fold) but significantly (t test, p<0.05) up-regulated by thiosulfate (Fig. 3C, D, Fig. S1). RpoE1 targets were not expressed at significantly lower levels in the Δ*rpoE1* or Δ*rpoE4* single mutant, whereas they were no longer up-regulated in the double Δ*rpoE1* Δ*rpoE4* mutant (Fig. 3C, D) showing that not only RpoE4, but also RpoE1, is activated by thiosulfate in exponential phase. This hypothesis is also supported by the observation that the *rpoE1-lacZ* fusion, although hardly induced after four hours in the presence of thiosulfate (<2-fold; Fig. 3D), was induced by thiosulfate after longer exposure, in an RpoE1 and RpoE4-dependent way (not shown). The overall low induction of RpoE1 targets suggests that RpoE1 is weakly activated by thiosulfate, and may explain why it did not complement the absence of RpoE4.

Microarray analyses of the *rpoE4*-overexpressing strain showed increased transcription of SMc00108, SMc04164 and SMc00821. However, these genes were not induced by thiosulfate (Fig. S2) nor did they display upstream putative RpoE4 or RpoE1 recognition sites. We concluded these are indirect effects of *rpoE4* overexpression. We assessed the transcriptomes of exponentially growing wt and Δ*rpoE4* mutant cells in the presence of thiosulfate, a true and unique activating condition for RpoE4 *vs* RpoE1, and found no significant differential expression of these genes (Table 2). We conclude that the RpoE4 regulon includes only the *rpoE4* and *sorT* operons.

### Activation of RpoE1 and RpoE4 in Stationary Phase Correlates with Endogenous Sulfite Accumulation during Growth

**Figure 5:**
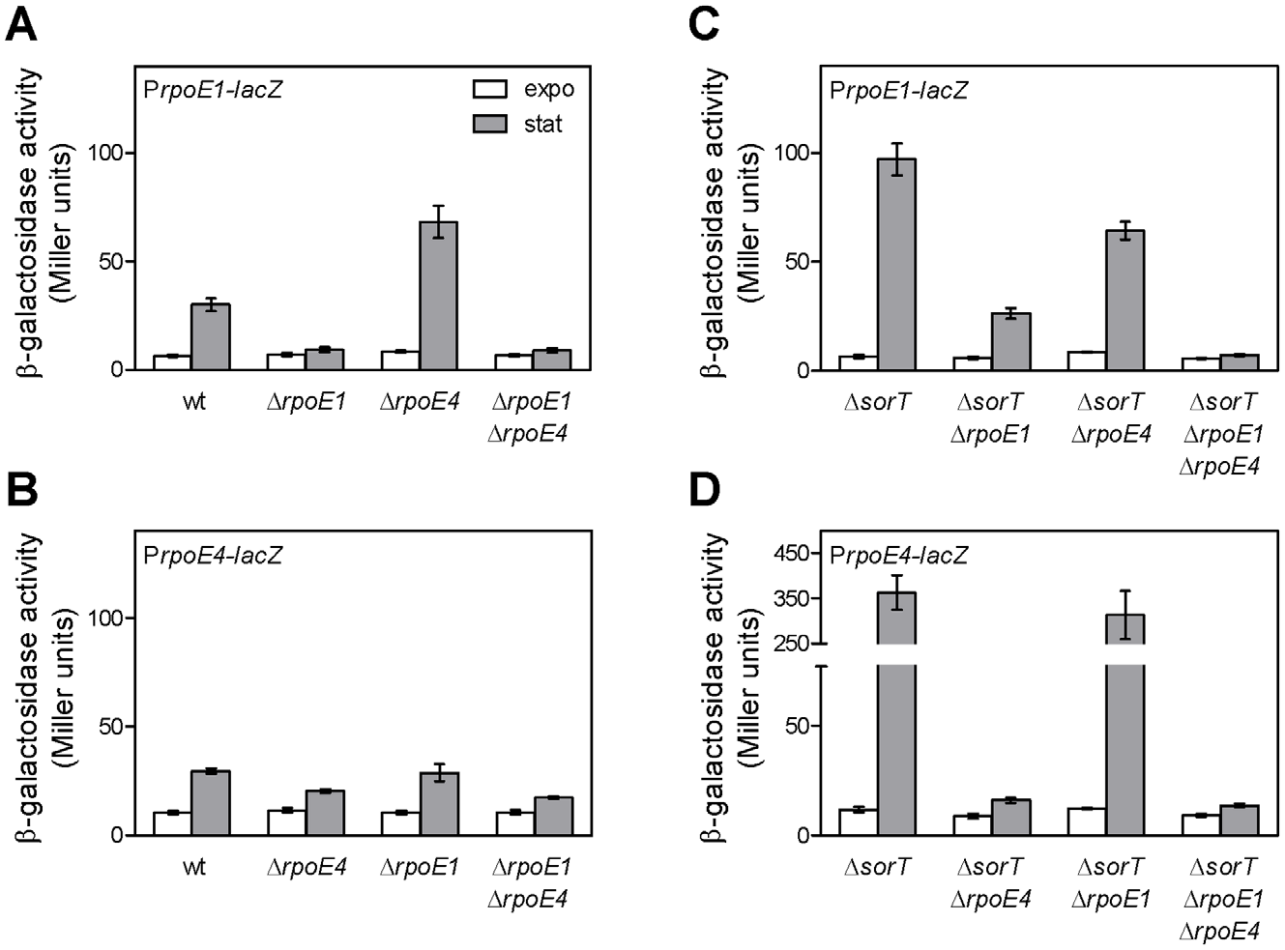
Expression of *rpoE1* and *rpoE4* at different growth phases and in various genetic backgrounds. Expression from the promoter of the *rpoE1* (A, C) or *rpoE4* (B, D) operon was estimated by measuring β-galactosidase activity driven from the chromosomal *PrpoE1-lacZ* fusion in strains CBT1183 (wt), CBT1185 (Δ*rpoE1*), CBT1191 (Δ*rpoE4*), (CBT1247 (Δ*rpoE1* Δ*rpoE4*), CBT1315 (Δ*sorT*), CBT1350 (Δ*sorT* Δ*rpoE1*), CBT1354 (Δ*sorT* Δ*rpoE4*) and CBT1358 (Δ*sorT* Δ*rpoE1* Δ*rpoE4*) or from the chromosomal *PrpoE4-lacZ* fusion in strains CBT1218 (wt), CBT1220 (Δ*rpoE1*), CBT1224 (Δ*rpoE4*), CBT1251 (Δ*rpoE1* Δ*rpoE4*), CBT1317 (Δ*sorT*), CBT1356 (Δ*sorT* Δ*rpoE4*), CBT1352 (Δ*sorT* Δ*rpoE1*) and CBT1360 (Δ*sorT* Δ*rpoE1* Δ*rpoE4*) grown in Vincent minimal medium with sodium succinate as carbon source either to exponential phase (OD_600_∼0.5; white bars) or stationary phase (∼24–30 h after the previous point; grey bars). The results shown are the means and standard errors of data from four to thirteen independent experiments.

We initially focused on RpoE1 and RpoE4 because they were pulled-down with RNAP in stationary phase. We wondered whether this reflected activation of RpoE1 and RpoE4 when cells transit from exponential to stationary phase. *rpoE1* and *rpoE4* promoter-*lacZ* transcriptional fusions were up-regulated ∼5 and ∼3-fold, respectively, in stationary *vs* exponential phase in the wt background (Fig. 5A and B), a finding in full agreement with recently published data [30]. Up-regulation of the *rpoE1-lacZ* fusion in stationary phase was abolished in the Δ*rpoE1* mutant (Fig. 5A), whereas that of the *rpoE4*-*lacZ* fusion was reduced in the Δ*rpoE4* mutant (Fig. 5A and B). These results, as well as other data presented below, demonstrate that both sigma factors RpoE1 and RpoE4 are activated in stationary phase.

We have shown above that in exponential phase, RpoE1 and RpoE4 can be activated by thiosulfate or taurine, and that both sigma factors control a sulfite oxidation response. A common feature of thiosulfate and taurine metabolisms is the generation of sulfite that could therefore be the actual stimulus for activation of RpoE1 and RpoE4. However, RpoE1 and RpoE4 activation in stationary phase was observed in minimal medium without addition of any known sulfite-generating compound. The following data suggest that RpoE1 and RpoE4 are activated in stationary phase by endogenous bacterial production of sulfite.

A first indication that bacteria endogenously generate sulfite came from the observation that expression of the *rpoE1-lacZ* fusion in stationary phase was more than 2-fold higher (t test, p<0.05) in the *rpoE4* mutant than in the wt strain, and that this expression was completely RpoE1-dependent (Fig. 5A). As the *rpoE4* mutation results in a decreased *sorT* expression and thus a lower sulfite oxidation (see above), these data suggested that RpoE1 was over-activated in the *rpoE4* mutant as a consequence of sulfite accumulation.

To confirm that *S. meliloti* generates sulfite during growth, we assayed the presence of sulfite in culture supernatants of cells grown to either exponential or stationary phase in minimal medium plus succinate as carbon source. Unfortunately, sulfite levels were around or below the detection threshold of the sulfite assay (∼1–1.5 µM) in cultures of the wt strain, presumably because of efficient sulfite oxidation. To increase the sensitivity of the test, we repeated it on cultures of the Δ*sorT* mutant, where sulfite should accumulate. Although sulfite could not be detected in culture supernatants of exponentially growing Δ*sorT* cells, a low but reproducible level of sulfite (2.7±0.6 µM) was detected in stationary phase, suggesting that sulfite is generated endogenously by *S. meliloti* during growth in this medium, and could therefore be responsible for RpoE1 and RpoE4 activation.

We therefore tested whether RpoE1 and RpoE4 activation in stationary phase correlates with endogenous sulfite levels, by comparing the stationary phase expression of the P*rpoE1-* and P*rpoE4-lacZ* fusions in wt and Δ*sorT* backgrounds. The fusions were expressed at levels ∼3 and 12-fold higher, respectively, in the Δ*sorT vs* wt background (Fig. 5C, D). As in the wt, induction of the fusions in the Δ*sorT* background was dependent on RpoE1 and RpoE4, respectively (Fig. 5C, D), showing that both sigma factors are activated under this condition. The observed over-activation of RpoE1 and RpoE4 in stationary phase in the *sorT* background was not due to a general, unspecific effect on sigma factors since in a control experiment, an RpoE2-dependent P*rpoE2-lacZ* fusion was up-regulated in stationary phase at similar levels in Δ*sorT* and wt backgrounds (not shown). Note that *rpoE1* expression was partly RpoE4-dependent in stationary phase in the *sorT* background, which provides another indication of the overlapping regulatory activities of RpoE1 and RpoE4 (Fig. 5C). Nevertheless, *rpoE4* expression in stationary phase was not detectably RpoE1-dependent, neither in the wt nor in the *sorT* background (Fig. 5B, D) which may be explained by a too weak activation of RpoE1 in this condition. Altogether, these data show that the level of sulfite which naturally accumulates endogenously during *S. meliloti* growth in minimal medium correlates with the RpoE1 and RpoE4 activation level. This suggests that sulfite could be one of the stimuli which directly or indirectly lead to RpoE1 and RpoE4 activation at the onset of stationary phase.

### RpoE1, RpoE4 and SorT are not Required for the *Medicago-S. meliloti* Symbiosis

*S. meliloti* is able to enter in symbiotic association with legume plants, in particular of the *Medicago* genera. In *Medicago* symbiotic root organs (nodules), most bacteria contained in infection threads as well as fully differentiated nitrogen-fixing bacteroids are non growing bacterial forms [11], [12] whose transcriptional activity resembles that of stationary phase of cultured bacteria [13]. Moreover, although it is not known whether legumes synthesize sulfite in response to rhizobial infection, sulfite was shown to be part of the antimicrobial defense in animals [42]. As RpoE1 and RpoE4 are activated in stationary phase, and more generally in the presence of sulfite, we wondered whether they could be involved in the establishment or functioning of the nitrogen-fixing symbiosis of *S. meliloti* with *Medicago* plants.

We therefore tested the symbiotic efficiency of Δ*rpoE1*, Δ*rpoE4*, Δ*rpoE1*Δ*rpoE4* and Δ*sorT* mutants, on *M. sativa* and/or *M. truncatula* (see Materials and methods). None of the mutants was affected in its nodule forming ability (Fig. S3) or its nitrogen fixation efficiency on nitrogen-free medium (as judged from the general state of the plant; not shown), showing that *rpoE1, rpoE4* and *sorT* are not essential for the *Medicago-S. meliloti* symbiosis.

## Discussion

### RpoE1 and RpoE4, two ECF Sigma Factors Belonging to the ECF26 Subgroup, are Activated in the Presence of Sulfite, and Display Overlapping Regulatory Activities

**Figure 6:**
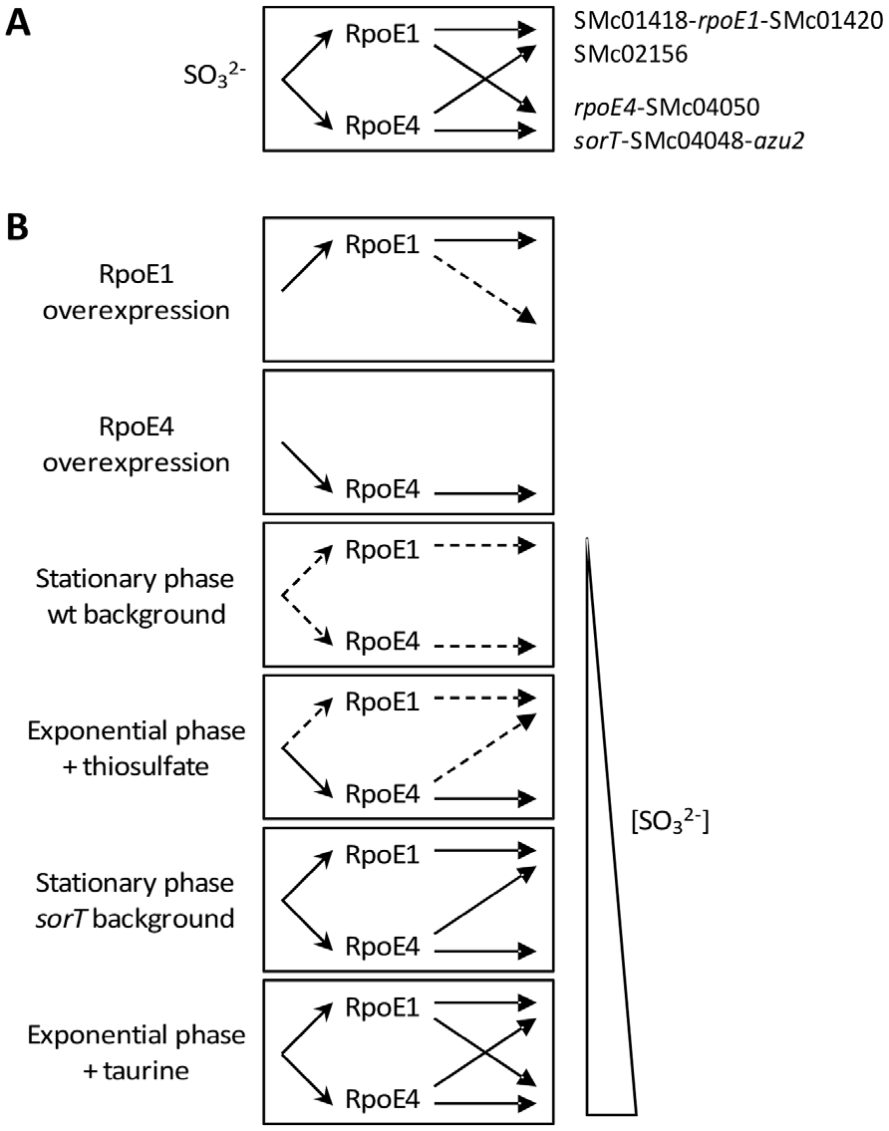
Model of gene regulation by RpoE1 and RpoE4 in *S. meliloti*. A. Sulfite (SO_3_^2−^) activates sigma factors (open arrowheads), which then control the transcription of target genes (closed arrowheads). The central box is repeated in part B. B. Interpretation of the data presented in this study. Under the different growth conditions tested (left), various levels of activation of the two sigma factors (indicated by no, dotted, or plain arrows) led to various levels of expression and cross-regulation of the target genes (indicated in the central box), and are interpreted as consequences of the various intracellular concentrations of sulfite present in the different conditions (right).

*S. meliloti* RpoE1, E3 and E4 sigma factors belong to the extracytoplasmic function (ECF) family, the largest and most diverse subfamily of sigma factors [43]. Recently, ECF sigma factors were classified into 43 major and 24 minor subgroups on the basis of sequence conservation of sigma factors and their putative anti-sigma factors, as well as of their genomic context [31]. Interestingly, RpoE1, E3 and E4, together with a fourth *S. meliloti* sigma factor (RpoE6) belong to the same major subgroup (ECF26; >100 members). Nothing was known to date about the function of these sigma factors, although Kappler and co-workers speculated that a member of this subgroup may be involved in regulation of a sulfite-oxidizing enzyme in response to thiosulfate [39], [44]. Staroń *et al*. [31] thus tentatively assigned “regulation of thiosulfate oxidation” as the putative function of ECF from this subgroup, although not experimentally supported. The data presented here suggest that a primary activating stimulus of RpoE1 and RpoE4 could be the presence of sulfite (SO_3_^2−^), and that both sigma factors control genes involved in sulfite oxidation (Fig. 6A). This work thus presents the first characterization of sigma factors from the ECF26 subgroup, and the first description of ECF sigma factors involved in sulfite metabolism. Nevertheless, preliminary data suggest that *rpoE3* and *rpoE6*, two other *S. meliloti* ECF26 encoding genes, are not induced by sulfite-generating compounds (not shown), suggesting that not all sigma factors from this subgroup are involved in sulfite metabolism.

Sulfite is naturally present in many environments, in particular those poor in oxygen, such as soil, the natural habitat of *S. meliloti*, where it is more stable than under aerobic conditions [45]. Sulfite is formed from sulfur dioxide, an environmental pollutant, but is also generated by bacteria either through assimilatory reduction of sulfate prior to biosynthesis of sulfur-containing amino acids [46], or through oxidation of more reduced inorganic or organic sulfur compounds, such as thiosulfate or taurine [47], [48], [49]. Taurine is present in the soil, and can be used by *S. meliloti* as carbon, energy and/or sulfur sources since all functions required for taurine transport and catabolism are encoded by *S. meliloti* (this study and [39], [50]). Sulfite is also known as a final product of the catabolism of sulfur-containing amino acids in eukaryotes [51]. Because of its high reactivity with biological macromolecules, sulfite is toxic to bacterial cells, which have thus evolved mechanisms to detoxify sulfite. Nevertheless, some bacterial species are able to use sulfite as a source of energy for growth [48], [52]. We found that RpoE1 and RpoE4 are activated in the presence of exogenously added thiosulfate or taurine, as well as in stationary phase, likely as a result of natural endogenous sulfite accumulation during growth. The origin of sulfite in the latter case is not known. We hypothesize that it could result from the catabolism of sulfur-containing molecules, such as the amino acids cystein and methionine, which may be used as alternative carbon sources upon exhaustion of succinate from the medium. Alternatively, sulfite produced as an intermediate of sulfate assimilation may accumulate upon entry into stationary phase.

RpoE1 and RpoE4 not only respond to the same stimulus (sulfite), but also display redundant regulatory activities, owing to overlapping recognition sequences in their target promoters (Fig. 6A). These shared features support their supposed common ancestral origin [31]. Such an overlap in the activating signals and regulated promoters was observed before in other bacteria encoding several ECF sigma factors, like *Bacillus subtilis* [53], [54]. The extent of regulatory overlap between RpoE1 and RpoE4 was dependent on the conditions tested, as summarized in Fig. 6B. Thus, in stationary phase (wt or *sorT* backgrounds) or in exponential phase in the presence of thiosulfate, no or little overlap was detected. In particular, RpoE1 did not control RpoE4 targets in these conditions, presumably because of a weak RpoE1 activation. In the presence of taurine, however, both sigma factors were strongly activated and were almost fully redundant. We assume that these progressive levels of activation result from i) the different susceptibility of activation of the sigma factors at a given sulfite concentration, RpoE4 being more sensitive to the presence of sulfite than RpoE1, and ii) the different sulfite concentrations present in various conditions (Fig. 6B). Thus, thiosulfate is likely a weaker sulfite donor than taurine, as supported by the fact that growth of the *rpoE4* mutant is not affected in the presence of thiosulfate, whereas it is severely reduced in the presence of taurine. Accordingly, no specific enzyme for thiosulfate assimilation is encoded by the *S. meliloti* genome [39]. We must say in this context that in spite of many attempts, we were not able to observe any significant induction of the *lacZ* reporter fusions described here upon direct addition of sodium sulfite to the cultures (up to 50 mM) even after long exposure (up to 24 hours, data not shown). We therefore assume that sulfite has to be generated inside the cells in order to activate the sigma factors.

### RpoE1 and RpoE4 Control a Sulfite Oxidation Response

RpoE1 and RpoE4 control a small number of genes, including their own operons, which contain a downstream gene encoding a putative anti-sigma factor protein (Fig. 1; [31]). Both auto-regulation and co-transcription with an anti-sigma factor-encoding gene are common properties of ECF sigma factors [31], [43].

The first gene of the *rpoE1* operon (SMc01418) encodes a putative secreted protein containing a repeated ‘lipoprotein 15′ motif of unknown function. Genes co-transcribed with ECF sigma factors often encode proteins involved in the regulation of sigma factor activity [43], and SMc01418 homologues are often encoded by *rpoE1*-like operons, even in phylogenetically distant bacteria. This suggests that SMc01418 may be involved in RpoE1 control. SMc02156 encodes a protein resembling periplasmic-binding proteins of ABC-type transport systems, whose best homologues are bacterial virulence-associated factors [55], [56], [57], although their precise function is unknown. Two transcriptomic reports described SMc02156 as highly expressed in the early stages of *S. meliloti*-*Medicago* symbiosis [13], [58] in which infecting bacteria resemble free-living bacteria in stationary phase [13], [58], [59], [60]. SMc02156 was also found to be transcriptionally induced in stationary phase in two different studies [13], [14]. SMc01418 and SMc02156 products were also detected in proteomic studies of either *Medicago* root nodules [61], [62], or free-living cultures where SMc01418 was more abundant in stationary phase, representing 10% of total proteins [30], [63]. Altogether, these data are in agreement with our finding that RpoE1 is most active in stationary phase. Strikingly, the SMc02156 product was found by Wilson and Kapler [39] as one of the major proteins co-purifying with SorT. Whether this reflects a true relationship with the sulfite-oxidizing enzyme, or simply that SMc02156 is an abundant contaminating protein in the conditions of SorT purification (i.e. culture in the presence of taurine) is presently unknown.

SorT is a dimeric molybdenum-containing sulfite dehydrogenase which catalyses oxidation of sulfite into sulfate [39], [52]. We showed here that SorT and its regulator RpoE4 are both needed for optimal growth of *S. meliloti* in a medium containing taurine as sole carbon source. We propose two hypotheses to explain this requirement. First, SorT may participate in sulfite detoxification. In its absence, residual growth may be allowed by the presence of three additional sulfite-oxidizing enzymes in *S. meliloti* [39]. Accordingly, we found that all three corresponding genes (SMa2103, SMb20584 and SMc01281) are up-regulated in the presence of taurine, both in the wt and the *rpoE1 rpoE4* mutant strains (not shown). Alternatively or in addition, sulfite oxidation by SorT, if coupled to the respiratory chain, may contribute to energy production and therefore significantly affect cell growth. Sulfite respiration was previously reported in another chemoheterotrophic bacterium, *Campylobacter jejuni* [64], and was suggested to occur in *S. meliloti* (cited as unpublished data in [39]). In this context, recent biochemical analyses suggested that the proteins encoded by the two genes forming an operon with *sorT*, a cytochrome *c* (SMc04048) and a pseudoazurin (SMc04047 or *azu2*), may function as acceptors for electrons generated from sulfite oxidation by SorT, and link it to the electron transport chain [40]. SorT is a periplasmic enzyme [39] indicating that sulfite has to transit via the periplasm in order to be oxidized. Although thiosulfate metabolism in *S. meliloti* is unknown, the desulfonation step of taurine catabolism takes place in the cytoplasm [50], [65]. The requirement for the periplasmic SorT for optimal growth on taurine as well as the presence of sulfite in culture supernatants implies that sulfite is exported from the cells. *S. meliloti* proteins involved in sulfite export are not known, but a gene of the taurine degradation locus was postulated to encode a putative sulfate transporter (SMb21531 or *tauZ*; [65]) that may be involved in sulfite export. In stationary phase, RpoE4 and SorT may provide the bacteria a means of sulfite detoxification, or contribute to energy production through sulfite respiration. In addition, sulfite was suggested to be part of the antimicrobial defense in animals [42]. It is not known whether legume plants synthesize sulfite in response to rhizobial infection, but the *S. meliloti* sulfite response is not essential for symbiosis with *Medicago*.

*sorT* expression is controlled by RpoE4, whose operon is located just upstream of *sorT*. Strikingly, in *Starkeya novella*, despite the absence of experimental evidence, Kappler and colleagues speculated that the *sorAB* genes, which encode a heterodimeric sulfite-oxidizing enzyme, were up-regulated in the presence of thiosulfate through the action of a sigma factor (RpoE) whose operon is located just upstream of *sorAB* (Fig. S4). Potential promoter sequences recognized by RpoE upstream of *sorA* were even predicted (although not experimentally tested) [44], [48] which almost perfectly match the *S. meliloti* RpoE4 recognition sequences (Fig. 4B). Interestingly, a similar genomic organization, i.e. the co-localization of genes encoding an ECF26 sigma factor, its putative anti-sigma factor and one or several putative enzymes involved in sulfite oxidation, is found in other α-Proteobacteria including another *S. meliloti* species (AK83), *S. medicae, Xanthobacter autotrophicus*, *Methylobacterium chloromethanicum, M. radiotolerans*, *Sulfitobacter* and *Roseovarius nubinhibens* (Fig. S4A). Moreover, promoter sequences similar to those recognized by RpoE4 could be found upstream of the sulfite-oxidase encoding genes in most of these bacteria (Fig. 4B). Finally, a similar genetic organization is present in β-Proteobacteria of the *Delftia*, *Comamonas* and *Acidovorax* genera (Fig. S4B), although in these cases the ECF sigma factor is more distantly related to RpoE4. Altogether, these observations suggest that mechanisms similar to those described in this study preside at transcriptional regulation of sulfite oxidizing enzymes in a wide range of bacteria.

## Supporting Information

Figure S1 ***rpoE1*** **and** ***rpoE4*** **are up-regulated in the presence of thiosulfate or taurine.** Expression levels of *rpoE1* and *rpoE4* were measured by qRT-PCR from strain GMI11495 (wt) either grown with sodium succinate (white bars) or taurine (pale grey bars) as carbon source, or with succinate plus 20 mM thiosulfate (dark grey bars). Results are expressed as relative transcript levels, with control levels arbitrarily set to 1 for each gene, and are the means and standard errors of data from three to five independent experiments.
(TIF)

Figure S2 **SMc00108, SMc04164 and SMc00881 are not up-regulated in the presence of thiosulfate.** Expression levels of SMc00108, SMc04164 and SMc00881 were measured by qRT-PCR from strain GMI11495 (wt) grown with sodium succinate as carbon source either in the absence (control, white bars) or in the presence (dark grey bars) of 20 mM thiosulfate. Results are expressed as relative transcript levels, with control levels arbitrarily set to 1 for each gene, and are the means and standard errors of data from three to four independent experiments.
(TIF)

Figure S3 **The** ***rpoE1*** **,** ***rpoE4*** **or** ***sorT*** **mutations do not affect the symbiotic capacity of** ***S. meliloti*** **.** Strains GMI11495 (wt), CBT1022 (Δ*rpoE1*), CBT997 (Δ*rpoE4*), CBT1064 (Δ*rpoE1* Δ*rpoE4*), or CBT1267 (Δ*sorT*) were used to inoculate roots of *Medicago sativa* (A) or *M. truncatula* (B) plantlets grown on nitrogen-free Fahraeus medium (time 0), and root nodules were numbered during ∼ 6 weeks. Each point represents the mean and standard error of data from 35–38 plants (*M. sativa*) in two independent experiments, or 10–11 plants (*M. truncatula*) in a single experiment. The *sorT* strain was tested on *M. sativa* in a single experiment (26 plants). In each experiment, a set of 10–11 plants was inoculated with sterile water as a negative control (H_2_O).
(TIF)

Figure S4 **Genomic organization of various α-(A) and β-proteobacteria (B) in regions encoding an ECF sigma factor and putative proteins involved in sulfite oxidation.** This drawing is a compilation of results from protein similarity searches using BlastP (http://blast.ncbi.nlm.nih.gov/Blast.cgi) and synteny searches using MaGe (https://www.genoscope.cns.fr/agc/microscope) and Absynte (http://archaea.u-psud.fr/absynte/). Genes which encode proteins with similar functions are depicted in the same colour (see legend), except unrelated genes which are represented in grey.
(TIF)

Table S1 **Oligonucleotides used in this study.**
(PDF)

Table S2 **Microarray expression data.**
(XLS)

Table S3 **List of proteins identified as associated with the** ***S. meliloti*** **RNAP in exponential and/or stationary phases of growth.**
(XLS)

Information S1 **Identification of sigma factors pulled down with** ***S. meliloti*** **RNAP in minimal medium.**
(PDF)
