# Supplementary figures and images for: Sinorhizobium meliloti Sigma Factors RpoE1 and RpoE4 Are Activated in Stationary Phase in Response to Sulfite
Source: PLoS One. 2012 Nov 30;7(11):e50768. doi: 10.1371/journal.pone.0050768 (PMC3511301; doi:10.1371/journal.pone.0050768)

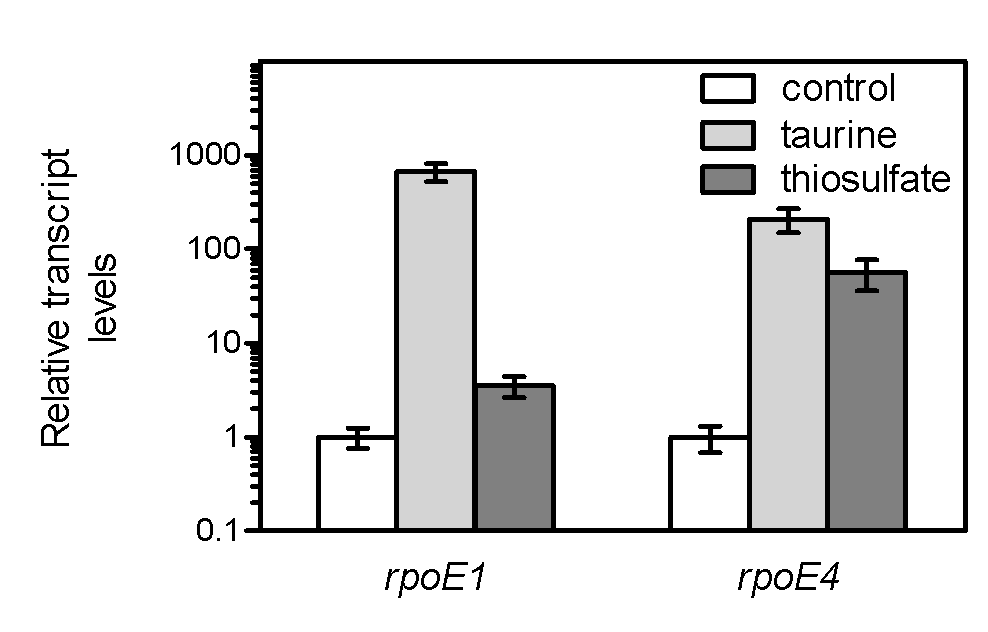

Supplement: Figure S1 — rpoE1 and rpoE4 are up-regulated in the presence of thiosulfate or taurine. Expression levels of rpoE1 and rpoE4 were measured by qRT-PCR from strain GMI11495 (wt) either grown with sodium succinate (white bars) or taurine (pale grey bars) as carbon source, or with succinate plus 20 mM thiosulfate (dark grey bars). Results are expressed as relative transcript levels, with control levels arbitrarily set to 1 for each gene, and are the means and standard errors of data from three to five independent experiments. (TIF) [file pone.0050768.s001.tif]

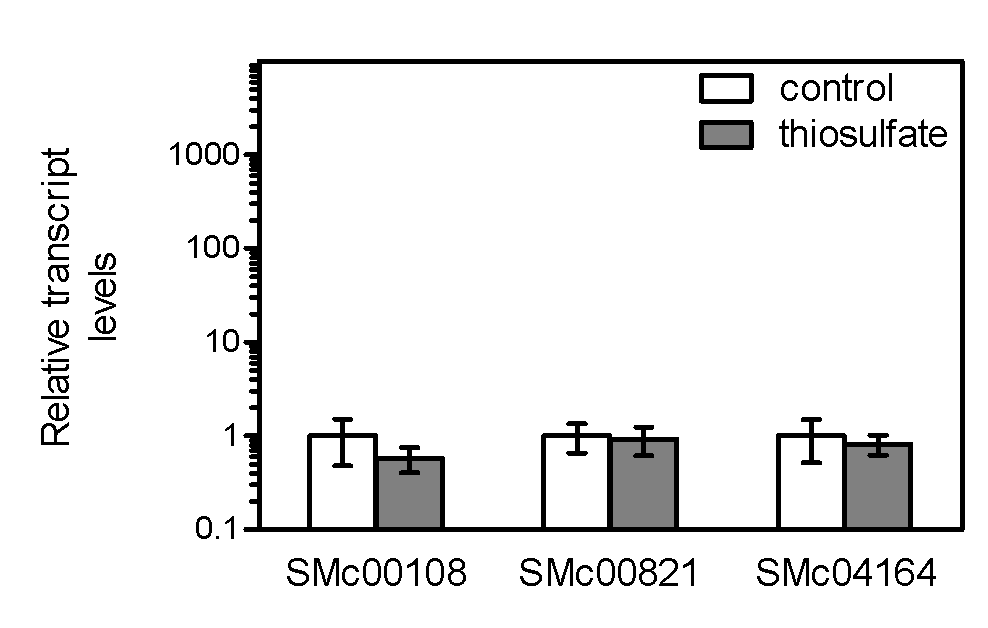

Supplement: Figure S2 — SMc00108, SMc04164 and SMc00881 are not up-regulated in the presence of thiosulfate. Expression levels of SMc00108, SMc04164 and SMc00881 were measured by qRT-PCR from strain GMI11495 (wt) grown with sodium succinate as carbon source either in the absence (control, white bars) or in the presence (dark grey bars) of 20 mM thiosulfate. Results are expressed as relative transcript levels, with control levels arbitrarily set to 1 for each gene, and are the means and standard errors of data from three to four independent experiments. (TIF) [file pone.0050768.s002.tif]

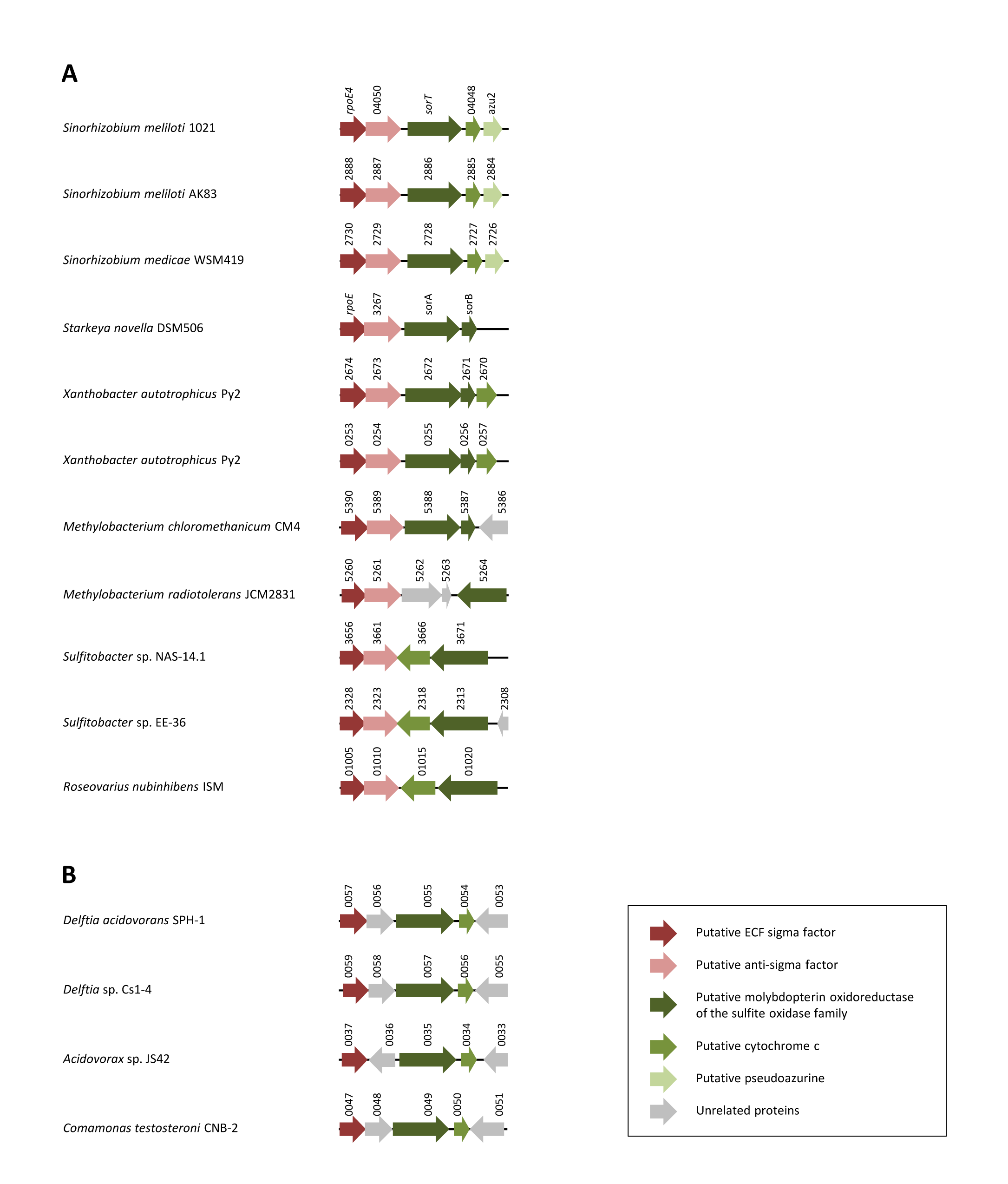

Supplement: Figure S4 — Genomic organization of various α-(A) and β-proteobacteria (B) in regions encoding an ECF sigma factor and putative proteins involved in sulfite oxidation. This drawing is a compilation of results from protein similarity searches using BlastP (http://blast.ncbi.nlm.nih.gov/Blast.cgi) and synteny searches using MaGe (https://www.genoscope.cns.fr/agc/microscope) and Absynte (http://archaea.u-psud.fr/absynte/). Genes which encode proteins with similar functions are depicted in the same colour (see legend), except unrelated genes which are represented in grey. (TIF) [file pone.0050768.s004.tif]
