# Supplementary material for: Sinorhizobium meliloti Sigma Factors RpoE1 and RpoE4 Are Activated in Stationary Phase in Response to Sulfite
Source: PLoS One. 2012 Nov 30;7(11):e50768. doi: 10.1371/journal.pone.0050768 (PMC3511301; doi:10.1371/journal.pone.0050768)
Supplement: Table S1 — Oligonucleotides used in this study. (PDF) [file pone.0050768.s005.pdf]

Table S2: oligonucleotides used in this study

Names	Sequences[1] (5'→ 3')	Main use
OCB588	ACGGCGAAGATCTGTTGCAG	qPCR *rpoE1* (SMc01419)
OCB589	GGTCATGATGGTGAAGGCCC	qPCR *rpoE1* (SMc01419)
OCB590	CAATGACGCCGACGATCTTG	qPCR *rpoE3* (SMc02713)
OCB591	TCGCATAGACCCAGCTTTCC	qPCR *rpoE3* (SMc02713)
OCB631	CGATCCAGATGTTCCGAAGG	Check pTH1703::*PrpoE4-lacZ* genomic insertion
OCB637	CCGCCAGGCAAATTCTG	Check insertions in pMLBAD
OCB638	ATTAGCGGATCCTACCTG	Check insertions in pMLBAD
DT88	GAAGAGAAGGTGGAAATGGCGTTTTGG	5' RACE
DT89	CCAAAACGCCATTTCCACCTTCTCTTC	5' RACE
OCB722	GCATGCAATAATTCTACACAGCCCAG	Pspac
OCB723	GCGGCCGCGGATCCGCGCTGGTACCCTCGCTC	Pspac
OCB724	CTCGAGTTTAAACTCATCATTTCCTTCCG	p*lacI*
OCB725	GCTAGCAATTGCGTTGCGCTCACTG	p*lacI*
OCB752	GGATCCACCTATATCGTCGGCGACAACG	*rpoC* upstream
OCB753	TCTAGATTCCGCCGCGGCGGGATCGTTCG	*rpoc* upstream
OCB755	GTTTAAACGCTTCCGCCTATCAGGGCGC	*rpoc* downstream
OCB758	GGCGACTACATCCTCGACGG	Check tag
OCB759	GGATAACGACAACGCACCCG	Check tag
OCB762	ATGCATGCGGAAAGGGGAGGGTATCTG	*rpoc* downstream
OCB794	GTTGGAATCGCTAGTAATCGCAG	qPCR 16S rDNA
OCB796	ACCTTCGGGTAGAACCAACTC	qPCR 16S rDNA
OCB907	GAATTCACC**ATG**TCGCACGGCCCTGACGG	*rpoE1* amplification
OCB908	AAGCTT**TCA**TTTCGGTCTCCGCAGGG	*rpoE1* amplification
OCB909	GTCGACCGTGCGCGGTTCTGCGTTGA	*rpoE1* upstream
OCB910	ATGCATGTCAGGGCCGTGCGACATCCC	*rpoE1* upstream
OCB911	ATGCATGCCCTGCGGAGACCGAAATG	*rpoE1* downstream
OCB912	CCCGGGGCATAGATCAGGAAAGCCGA	*rpoE1* downstream
OCB915	TCTAGAGCTCCACTGGCTCAAGGGGC	*rpoE3* upstream
OCB916	ATGCATTGCTCTTCCAGTGAAACGTG	*rpoE3* upstream
OCB919	GAATTCACC**ATG**TCGGGTATCATGCGCTC	*rpoE4* amplification
OCB920	AAGCTT**TCA**TTTTCGGCCTTCTCGAT	*rpoE4* amplification
OCB921	GTCGACGCTCGCCATGGATCGCACCA	*rpoE4* upstream and P-*rpoE4*
OCB922	ATGCATGACACCCGATCAGGGCTGCA	*rpoE4* upstream and P-*rpoE4*
OCB923	ATGCATGCGCGGCATCGAGAAGGCCG	*rpoE4* downstream
OCB924	CCCGGGCGGCGTGAAAGAACCTCCGA	*rpoE4* downstream
OCB935	CGGTTCTCGGTGTTGCGGCC	Check *rpoE1* deletion
OCB939	GGACCAGCCGCGCGGAAAAC	Check *rpoE1* deletion
OCB942	CCTGTGCCTGCATGTCCAGC	Check *rpoE4* deletion
OCB943	CAGGCTCAGCCGACAGCCGG	Check *rpoE4* deletion
OCB950	ATTCGATCGTTGAGCGAAAG	qPCR SMc01418
OCB951	CGGTAACGTCACCCATCTTC	qPCR SMc01418
OCB960	GACCGATCGTTCCTTGGATAG	P-SMc01418-*rpoE1*
OCB961	GGGTTTCCTCCTCAAGATGTC	P-SMc01418-*rpoE1*

OCB964	TGATCTATGCCAGCGAAGTG	qPCR SMc01420
OCB965	AAAGATCGGGGATTTTCAGC	qPCR SMc01420
OCB966	AGGATACCGCACTCCATGAC	qPCR SMc02156
OCB967	TCTCCTTCGTCCAGACATCC	qPCR SMc02156
OCB968	ATGAACGAGCATTCGTCATC	qPCR SMc04164
OCB969	GAGAGGTTCTCCAGGACACG	qPCR SMc04164
OCB970	ACTGTCGCCGAACTGAAGAC	qPCR SMc04049
OCB971	ATTTTCTGGTCACCCGTCAG	qPCR SMc04049
OCB976	TTGATCGGTTATGCCATTCTC	qPCR SMc00108
OCB977	ACGAAGAGGTGGTGCATTTC	qPCR SMc00108
OCB982	ACCTGGAACAGGAGTTCGTC	qPCR *rpoE4* (SMc04051)
OCB983	AAACCGGATATGTCGACCAG	qPCR *rpoE4* (SMc04051)
OCB991	TCTAGACGTTGCATACAGGCTACGTC	*sorT* upstream
OCB992	ATGCATGTCGCGTCTCCTTTTCCC	*sorT* upstream
OCB993	ATGCATCCGCAACCGAAGGGTTCC	*sorT* downtream
OCB994	CCCGGGGCCGATCACTTGGCTTTTC	*sorT* downtream
OCB995	CTTTTGCGACGAAGGTGACG	Check *sorT* deletion
OCB996	CTGGGTCGGAGGTTCTTTC	qPCR SMc04050
OCB997	CAAAGACGGGCGTCAATC	qPCR SMc04050
OCB998	GGTACC**TTG**ACTGAGATGCTGACCAT	*sorT* amplification
OCB999	GCATGC**TCA**GGCAACGGTGAGTTTGA	*sorT* amplification
OCB1002	GTTTGAAACGCGAAGACCTG	qPCR SMc00821
OCB1003	TCCCCTGTAGAAGAGGTTCG	qPCR SMc00821
OCB1004	GATGGATGCTGTCGTTTGCTG	Check *sorT* deletion
OCB1042	GAGGATGCGGTGCATATCCTC	Check pTH1703::*PrpoE1-lacZ* genomic insertion
OCB1050	TCCGTATCCTGCGCAATCTC	qPCR *rpoE6* (SMa0143)
OCB1051	GATGAACCGGCGATGTCATG	qPCR *rpoE6* (SMa0143)
OCB1136	CTCTGGCGGGAGCCGTTG	qPCR SMb21671
OCB1137	GGACACGACAAGCGGCTTG	qPCR SMb21671
KS	TCGAGGTCGACGGTATC	Check insertions in pJQ200KS
SK	CGCTCTAGAACTAGTGGATC	Check insertions in pJQ200KS
Gus1	TGCCCACAGGCCGTCGAG	Check pTH1703 derivatives genomic insertion
Gus Rev	TAACGCGCTTTCCCACCAACGCTGATCA	Check insertions in pTH1703
lacPCR	AAGGGGGATGTGCTGCAAGG	Check insertions in pTH1703
M13 -20	GTAAAACGACGGCCAGT	Check insertions in pJQ200mp19 and pGEM-T
M13 Rev	GGAAACAGCTATGACCAT	Check insertions in pJQ200mp19 and pGEM-T

[1]Bold letters represent start and stop codons. Underlined or highlighted sequences indicate restriction sites.
