# Supplementary material for: Sinorhizobium meliloti Sigma Factors RpoE1 and RpoE4 Are Activated in Stationary Phase in Response to Sulfite
Source: PLoS One. 2012 Nov 30;7(11):e50768. doi: 10.1371/journal.pone.0050768 (PMC3511301; doi:10.1371/journal.pone.0050768)
Supplement: Information S1 — Identification of sigma factors pulled down with S. meliloti RNAP in minimal medium. (PDF) [file pone.0050768.s008.pdf]

Supporting information S1

Identification of sigma factors pulled down with *S. meliloti* RNAP in minimal medium

Activation of a sigma factor results in its interaction with RNAP. We hypothesised that identifying proteins co-purifying with RNAP in stationary phase would make possible to discover the sigma factors active in this condition. We thus used a tandem affinity purification approach to isolate *S. meliloti* RNAP. A strain expressing a tagged form of the β' subunit of RNAP at wild type (wt) levels was generated by introduction of a 217 bp extension encoding the SPA-tag at the 3' end of the native chromosomal copy of the *rpoC* gene. The resulting strain (CBT789) grew as efficiently as the wt strain (GMI11495) at 28°C in either rich LBMC medium or Vincent minimal medium supplemented with sodium succinate as carbon source. This indicates that the tagged RNAP efficiently interacts with the vegetative sigma factor RpoD as well as with the alternative sigma factor RpoN required for succinate utilisation [1]. An RpoE2-dependent promoter-*lacZ* reporter fusion was also found to be inducible following a heat shock in this strain (not shown), demonstrating that the tagged RNAP is able to interact with the ECF sigma factor RpoE2. Altogether, these observations indicated that the tagged RNAP was fully active and able to interact with both vegetative and alternative sigma factors.

rpoC-SPA cells grown in minimal medium to either exponential or early stationary phase were used to isolate RNAP by tandem affinity purification. As a control, the same treatment was applied to wt (untagged) *S. meliloti* cells. Purified proteins were separated by SDS-PAGE, and coomassie staining revealed a number of discrete bands in lanes corresponding to the *rpoC-spa* samples, as expected, whereas no band was visible from the control samples. As no qualitative or quantitative difference was visible between the two *rpoC-spa* samples, gel lanes were sliced, proteins were in-gel digested with trypsine and subjected to liquid chromatography coupled to tandem mass spectrometry (LC-MS/MS). The whole experiment was repeated twice independently, and only proteins represented in both repeats of each condition were kept for further analysis. A complete list of the proteins identified and validated with a false discovery rate < 1% is given in Table S3.

As expected, the α, β, β' and ω subunits of the core RNAP were present in both exponential and stationary phases. A number of cellular proteins co-purified with RNAP in all experiments, including ribosomal proteins, translation elongation factors, protein chaperones and proteases; a majority of these proteins were also found by others in similar experiments performed on different bacteria [Table S3 and 2,3,4,5] and were not further considered. Among proteins known to associate with RNAP, the vegetative sigma factor RpoD and the accessory proteins NusA, NusG and Rho were found in both exponential and stationary phases, as expected [6,7,8] as well as the heat shock sigma factor RpoH1. To find RpoH1 active at 28°C is not surprising since *S. meliloti rpoH1* mutant cells display a slight growth defect at 30°C [6] and RpoH1 controls the transcription of some genes at 30°C [8,9]. For unknown reasons, we did not isolate RpoN, which indicates that some sigma factors can be missed by this approach. Interestingly, five sigma factors (RpoE2, RpoH2, RpoE1, RpoE3 and RpoE4) were found to co-purify with RNAP in stationary phase only, although we cannot exclude that these sigma factors were not detected in exponential phase because they were under the detection threshold. The presence of RpoE2 and RpoH2 in stationary phase was not surprising, as both are activated in this growth phase [6,9,10]. The *rpoE1, rpoE3* and *rpoE4*

genes were recently described as up-regulated in stationary phase [11], but little was known about the function of these sigma factors and the conditions in which they are activated. In this work, we report a functional characterization of RpoE1 and RpoE4.

Materials and methods

Construction of the *rpoC-SPA* strain CBT789

The strain CBT189, which carries the chromosomal *rpoC-SPA* fusion, was constructed as follows. The plasmid pJQ200KS-SPA was first constructed by cloning into pJQ200KS the Pspac promoter, the SPA-tag and the *lacI* gene of pMUTIN-SPA (SPA-tag delivery plasmid [12]), and the *rrnB* terminator of pMLBAD (construction details and DNA sequence available upon request). ~400 bp regions flanking the *rpoC* stop codon were amplified by PCR using OCB752-753 and OCB762-755 as primers, and individually cloned into pGEM-T. These regions were then successively inserted on either side of the SPA-tag in pJQ200KS-SPA as *Pme*I/*Sal*I and *Bam*HI/*Xba*I fragments, respectively, thus generating pJQ200KS-*rpoC-SPA*. The strain was constructed as described for the deletion mutants in the main text. Insertion of the SPA tag was screened by PCR using primers OCB758-OCB759.

RpoC-SPA purification

Cells were grown at 28°C with aeration in VMMS supplemented with 100 µg ml^{-1} Sm in 2 L flasks. 200ml of exponential phase culture (OD_{600} ~ 0.6) or early stationary phase culture (1h30 after entry into stationary phase; OD_{600} ~1.2) were harvested by centrifugation at 4°C. Cells were washed twice with 80 ml and 40 ml of buffer A (10mM Tris-HCl pH 7.5; 150mM NaCl). Pellets were frozen in liquid nitrogen and stored at -80°C.

All following steps were performed at 4°C. Bio-spin columns (Bio-Rad) were used. To lyse cells, pellets were thawed, re-suspended in Bug Buster Master Mix (Novagen) and incubated for ~45 min at room temperature. Cell lysates were sonicated and centrifuged for 30min at 20.000g. Equal amounts of proteins from control and assay samples, estimated by OD_{280}, were incubated for 3h on a rocking shaker (Apelex, 10 oscillations min^{-1}) with 100µl of anti-FLAG M2 agarose beads (Sigma Aldrich) washed with buffer B (10mM Tris-HCl pH7.5, 150mM NaCl, 0.2mM EDTA, 0.1% Triton 100X, 1mM DTT). Supernatant was removed and the anti-FLAG resin was washed 5-times with buffer B on a Poly-prep column. Beads were incubated over-night in 200µl TEV buffer (50 mM Tris-HCl pH 7.5, 150 mM NaCl, 0.2 mM EDTA, 0.1% Triton X-100, 1 mM DTT) containing 25 units of TEV protease. The eluate was collected on a second bio-spin column and beads were washed once with 400µl of TEV buffer. 100µl of calmoduline-sepharose beads (Amersham biosciences) previously washed with Calmoduline Binding Buffer (CBB; 10 mM Tris-HCl pH 7.5, 150 mM NaCl, 2 mM $CaCl_2$, 0.1% Triton X-100, 10 mM 2-mercaptoethanol) and 5µl of 240 mM $CaCl_2$ was added to the eluate and incubated for 3h with agitation. Beads were washed twice with 250µl of CBB buffer and once with 100µl of Calmoduline Wash Buffer (CWB; 10 mM Tris-HCl pH 7.5, 150 mM NaCl, 0.1 mM $CaCl_2$, 0.1% Triton X-100, 10 mM 2-mercaptoethanol). Elution was performed with 250µl of Calmoduline Elution Buffer (CEB; 10 mM Tris-HCl pH 7.5, 150 mM NaCl, 3 mM EGTA, 10 mM 2-mercaptoethanol). An additional step of elution was performed with 250µl of CEB buffer incubated for 15 min with beads. 3 volumes of acetone were added to the eluate, incubated for 3h at -20°C and centrifuged for 30 min at 12.000g.

The supernatant was removed and pellets were dried ~15min at 37°C. Proteins were resuspended in Laemmli buffer, reduced by incubation for 30 min at 56°C with 30mM DTT (Euromedex, EU0006-D) and then alkylated by incubation for 30 min at room temperature with 90mM iodoacetamide (Sigma-I6125). Half of the eluate was loaded on a 12% SDS-PAGE and run until half of the gel was reached. The gel was stained with Page Blue protein staining solution (Fermentas). Each gel lane was systematically cut into 10 bands of similar volume for MS/MS protein identification. Each band was incubated in 25 mM ammonium bicarbonate and 50% ACN until destaining. Gel pieces were dried in a vacuum SpeedVac (45°C), further rehydrated with 30 µl of a trypsin solution (10 ng l^{-1} in 50mM NH_4HCO_3), and finally incubated overnight at 37°C. The resulting peptides were extracted from the gel as described previously [13]. The trypsin digests were dried in a vacuum SpeedVac and stored at -20 °C before LC-MS/MS analysis.

Nano-LC-MS/MS Analysis

For MS analysis, tryptic peptides were resuspended with 12 µl of 5% ACN, 0.05% TFA and were submitted to nano-LC-MS/MS using an Ultimate3000 system (Dionex, Amsterdam), coupled to an LTQ-Orbitrap mass spectrometer (Thermo Fisher Scientific, Bremen, Germany). Five microliters of each digest were loaded on a C18 precolumn (300µm inner diameter X 5 mm; Dionex) at 20 µl min^{-1}. After 5 min of desalting, the precolumn was switched on line with the analytical column (75µm inner diameter X 15 cm; PepMap C18, Dionex) equilibrated in 95% solvent A (5% ACN, 0.2% formic acid) and 5% solvent B (80% ACN, 0.2% formic acid).Peptides were eluted using a 5–50% gradient of solvent B during 80 min at 300 nl min^{-1} flow rate. The LTQ-Orbitrap was operated in data-dependent acquisition mode with the Xcalibur software (version 2.0.6, Thermo Fisher Scientific). Survey scan MS spectra were acquired in the Orbitrap on the 300–2000 *m/z* range with the resolution set to a value of 60,000. The five most intense ions per survey scan were selected for CID fragmentation, and the resulting fragments were analyzed in the linear trap (LTQ). Dynamic exclusion was used within 60 s to prevent repetitive selection of the same peptide. To automatically extract peak lists from Xcalibur raw files, the ExtractMSN macro provided with Xcalibur was used through the Mascot Daemon interface (version 2.3.2, Matrix Science, London, UK). The following parameters were set for creation of the peak lists: parent ions in the mass range 400–4500, no grouping of MS/MS scans, and threshold at 1000. A peak list was created for each fraction analyzed (*i.e.* gel slice), and individual Mascot searches were performed for each fraction.

Database search and validation of results

The data were searched against the protein database of *S. meliloti* (UniProtKB) containing 6169 protein sequences. Mass tolerances in MS and MS/MS were set to 5 ppm and 0.8 Da, respectively, and the instrument setting was specified as “ESI Trap”. Trypsin (specificity set for cleavage after K or R) was designated as protease, and one missing cleavage was allowed. Oxidation of methionine was searched as variable modification, and carbamidomethylation of cysteine was set as fixed modification. The MFPAQ software [14] was used to filter the MASCOT results. Each protein was validated according to computer-defined criteria with a FDR (False Discovery Rate) limited to 1%.

References

1. Ronson CW, Nixon BT, Albright LM, Ausubel FM (1987) *Rhizobium meliloti ntrA* (*rpoN*) gene is required for diverse metabolic functions. J Bacteriol 169: 2424-2431.
2. Butland G, Peregrin-Alvarez JM, Li J, Yang W, Yang X, et al. (2005) Interaction network containing conserved and essential protein complexes in *Escherichia coli*. Nature 433: 531-537.
3. Pelletier DA, Hurst GB, Foote LJ, Lankford PK, McKeown CK, et al. (2008) A general system for studying protein-protein interactions in Gram-negative bacteria. J Proteome Res 7: 3319-3328.
4. Hervey WJt, Khalsa-Moyers G, Lankford PK, Owens ET, McKeown CK, et al. (2009) Evaluation of affinity-tagged protein expression strategies using local and global isotope ratio measurements. J Proteome Res 8: 3675-3688.
5. Delumeau O, Lecointe F, Muntel J, Guillot A, Guedon E, et al. (2011) The dynamic protein partnership of RNA polymerase in *Bacillus subtilis*. Proteomics 11: 2992-3001.
6. Oke V, Rushing BG, Fisher EJ, Moghadam-Tabrizi M, Long SR (2001) Identification of the heat-shock sigma factor RpoH and a second RpoH-like protein in *Sinorhizobium meliloti*. Microbiology 147: 2399-2408.
7. Roberts JW, Shankar S, Filter JJ (2008) RNA polymerase elongation factors. Annu Rev Microbiol 62: 211-233.
8. de Lucena DK, Puhler A, Weidner S (2010) The role of sigma factor RpoH1 in the pH stress response of *Sinorhizobium meliloti*. BMC Microbiol 10: 265.
9. Barnett MJ, Bittner AN, Toman CJ, Oke V, Long SR (2012) Dual RpoH sigma factors and transcriptional plasticity in a symbiotic bacterium. J Bacteriol.
10. Sauviac L, Philippe H, Phok K, Bruand C (2007) An extracytoplasmic function sigma factor acts as a general stress response regulator in *Sinorhizobium meliloti*. J Bacteriol 189: 4204-4216.
11. Barra-Bily L, Fontenelle C, Jan G, Flechard M, Trautwetter A, et al. (2010) Proteomic alterations explain phenotypic changes in *Sinorhizobium meliloti* lacking the RNA chaperone Hfq. J Bacteriol 192: 1719-1729.
12. Lecointe F, Serena C, Velten M, Costes A, McGovern S, et al. (2007) Anticipating chromosomal replication fork arrest: SSB targets repair DNA helicases to active forks. Embo J 26: 4239-4251.
13. Borderies G, Jamet E, Lafitte C, Rossignol M, Jauneau A, et al. (2003) Proteomics of loosely bound cell wall proteins of *Arabidopsis thaliana* cell suspension cultures: a critical analysis. Electrophoresis 24: 3421-3432.
14. Bouyssié D, Gonzalez de Peredo A, Mouton E, Albigot R, Roussel L, et al. (2007) Mascot file parsing and quantification (MFPaQ), a new software to parse, validate, and quantify proteomics data generated by ICAT and SILAC mass spectrometric analyses: application to the proteomics study of membrane proteins from primary human endothelial cells. Mol Cell Proteomics 6: 1621-1637.
